# Genome-wide multi-omics profiling of colorectal cancer identifies immune determinants strongly associated with relapse

**DOI:** 10.3389/fgene.2013.00236

**Published:** 2013-11-20

**Authors:** Subha Madhavan, Yuriy Gusev, Thanemozhi G. Natarajan, Lei Song, Krithika Bhuvaneshwar, Robinder Gauba, Abhishek Pandey, Bassem R. Haddad, David Goerlitz, Amrita K. Cheema, Hartmut Juhl, Bhaskar Kallakury, John L. Marshall, Stephen W. Byers, Louis M. Weiner

**Affiliations:** ^1^Department of Oncology, Innovation Center for Biomedical Informatics, Georgetown University Medical CenterWashington, DC, USA; ^2^Department of Oncology, Lombardi Comprehensive Cancer Center, Georgetown University Medical CenterWashington DC, USA; ^3^Indivumed GmbHHamburg, Germany; ^4^Department of Pathology, Lombardi Comprehensive Cancer Center, Georgetown University Medical CenterWashington DC, USA

**Keywords:** colorectal cancer, relapse, variant analysis, integrative analysis, multi-omics, exome sequencing, systems biology, immune response

## Abstract

The use and benefit of adjuvant chemotherapy to treat stage II colorectal cancer (CRC) patients is not well understood since the majority of these patients are cured by surgery alone. Identification of biological markers of relapse is a critical challenge to effectively target treatments to the ~20% of patients destined to relapse. We have integrated molecular profiling results of several “omics” data types to determine the most reliable prognostic biomarkers for relapse in CRC using data from 40 stage I and II CRC patients. We identified 31 multi-omics features that highly correlate with relapse. The data types were integrated using multi-step analytical approach with consecutive elimination of redundant molecular features. For each data type a systems biology analysis was performed to identify pathways biological processes and disease categories most affected in relapse. The biomarkers detected in tumors urine and blood of patients indicated a strong association with immune processes including aberrant regulation of T-cell and B-cell activation that could lead to overall differences in lymphocyte recruitment for tumor infiltration and markers indicating likelihood of future relapse. The immune response was the biologically most coherent signature that emerged from our analyses among several other biological processes and corroborates other studies showing a strong immune response in patients less likely to relapse.

## Introduction

Colorectal cancer (CRC) is the third most commonly diagnosed cancer in the United States in both men and women. In 2013, an estimated 142,820 new cases will be diagnosed, and 50,830 deaths from CRC are expected to occur in the United States (ACS, 2013). Great effort is being made to identify molecular signatures in CRC that both serve as prognostic markers of recurrence, and that allow for identification of subgroups of patients who would benefit from a particular chemotherapy. Equally important is the identification of patients who might not benefit from particular treatments based on their disease stage and molecular profile, in an effort to spare them unnecessary toxicity.

Standard treatment for stage III colon cancer includes adjuvant chemotherapy after surgery, which results in improvement in progression-free and overall survival compared to surgery alone (Schrag et al., 2001). However, a lower recurrence rate after surgery makes the benefits of adjuvant therapy for earlier stage CRC less clear (Chau and Cunningham, 2006). Virtually all stage I colon cancers, and approximately 80% of stage II colon cancer patients are cured by appropriate surgery (Benson, 2006; Lavery and De Campos-Lobato, 2010) however, 20% of stage II patients relapse, and many of them will die due to metastatic disease. Adjuvant chemotherapy has no role in stage I and little or no impact on relapse or overall survival in stage II colon cancer, although there is a significant increase in disease-free survival after therapy (Figueredo et al., 2008). Therefore, in early stage colon cancer the benefits of adjuvant therapy must be weighed against the risks of toxicity for 80% (higher in stage I) of the target population that has been cured by surgery, and in consideration of poor enhancement of overall survival in the relapse group for stage II CRC patients. The challenge for personalized early stage colon cancer treatment is to identify clinical or molecular determinants of outcome in order to target treatments to those individuals who are destined to relapse.

Personalized cancer treatment requires comprehensive genetic information of individual cancers. While isolated analysis of genomic data types are of clinical value, an integrated and comprehensive analysis of multiple genomic data types from individual cancers leverages the predictive power of each data type and allows for an understanding of the complex molecular networks that drive tumor behavior at systemic level. Such information is extremely valuable in not only developing therapeutic strategies, but also predicting tumor response to specific treatment modalities for individual cancers. Based on these predictions, target patients may be identified and segregated into those who may benefit and those not likely to benefit from a particular therapy, and therefore be spared of “pain without gain.” The patient community would be well served by the identification of effective prognostic biomarkers in the serum or urine that could be used to supplement the most common mechanism of prognostication which is the AJCC tumor, node and metastases (TNM) staging classification. The existence of a noninvasive method such as analysis of serum and urine to help diagnose the extent of disease or predict outcome would likely result in significant improvements in patient response by enabling much earlier, and more cost-effective prognosis. Also, whole genome profiling is not always feasible in a clinical setting and there is a need for a small set of the most informative markers that can predict outcome and response to therapies. We postulated that a multi-dimensional molecular analysis of tumors followed by rigorous bioinformatics analysis will yield a combination of features that serve as prognostic biomarkers of relapse in stage II and stage I adenocarcinoma of the colon.

Several molecular approaches are being used to identify patients who may benefit from adjuvant chemotherapy due to a higher risk for relapse. The most common methods include: gene expression analysis for biomarker identification, immunohistochemical assays for aberrant protein expression, chromosomal and microsatellite instability (MSI) detection to find mutation hotspots, and identifying gene variants through analysis of single nucleotide polymorphisms. The objectives of our study were to first use multi-omics molecular profiling data and to integrate several data types using classification algorithms and multivariate analysis to determine the “molecular portrait” of relapse; and second, to use systems biology tools to elucidate functional modules, cellular processes and pathways that are most affected and strongly associated with CRC relapse.

Many investigations have uncovered several critical genes and pathways such as WNT, RAS2MAPK, PI3K, TGF-b, P53, and DNA mismatch-repair pathways that are important in the initiation and progression of CRC (Fearon, 2011). Multiple sequencing analyses studies have identified numerous recurrently mutated genes (TCGA, 2012). Attempts to correlate clinical outcome with molecular signatures are usually confined to analysis of one data type, for instance prediction of outcome in stage II CRC patients with 4q deletions (Brosens et al., 2010), methylation levels of specific MINT loci as prognostic variables in patients with stage I and II rectal cancers (de Maat et al., 2008), and the 12-gene recurrence score gene expression study by CALGB (Venook et al., 2013). Despite these advances, we do not have a fully integrated view of the genetic and genomic changes and their significance for colorectal relapse. This is especially important in light of the single clinically applicable genomic information that is used—the KRAS status, where mutations predict lack of efficacy of EGFR antibodies. Stage II CRC patients thus would benefit more from the identification of better prognostic and predictive markers. Such clinically useful biomarkers also could provide insights into the biology behind recurrence, which may further help to target the relevant pathways.

It is well established that the development of cancer is associated with alterations in immune cells in the peripheral circulation and also at the sites of tumor progression and metastasis. Recently, a possibility has emerged that immune measures such as tumor infiltrating lymphocytes (TILs) could serve as biomarkers or as surrogate endpoints of clinical outcome or responses. Progress in our understanding of cellular and molecular pathways involved in immune responses to cancer has greatly facilitated the selection of the most relevant immune endpoints to discover and evaluate (Pages et al., 2005; Galon et al., 2006; Whiteside, 2013). To fully elucidate genetic underpinnings of colorectal relapse, a systems biology approach is necessary to characterize variants, mRNA, copy number, and metabolites, as well as their interactions within the cells (Rodin et al., 2011) as well as with other cells such as those from the immune system within the tumor microenvironment. Gene set and pathway association analyses are playing an increasingly important role in explaining disease mechanisms through the identification of functional genetic interactions (Rodin et al., 2011). An integrative approach combining multiple data types can more accurately capture pathway associations clinically actionable variants.

In this pilot study we integrated the results of molecular profiling of several omics data types to determine the most reliable prognostic molecular signature for relapse in CRC. The data types were integrated using multi-step analytical approach with consecutive elimination of redundant molecular features. As a result a minimum number of most informative multi-omics features were determined allowing for the best classification accuracy of relapse phenotype. Taken together these data show that biomarkers detected in the tumors, urine and blood of patients collected at the time of surgery point to a strong association of immune processes and markers with likelihood of future relapse.

## Results

### Analysis overview

**Figure 1:**
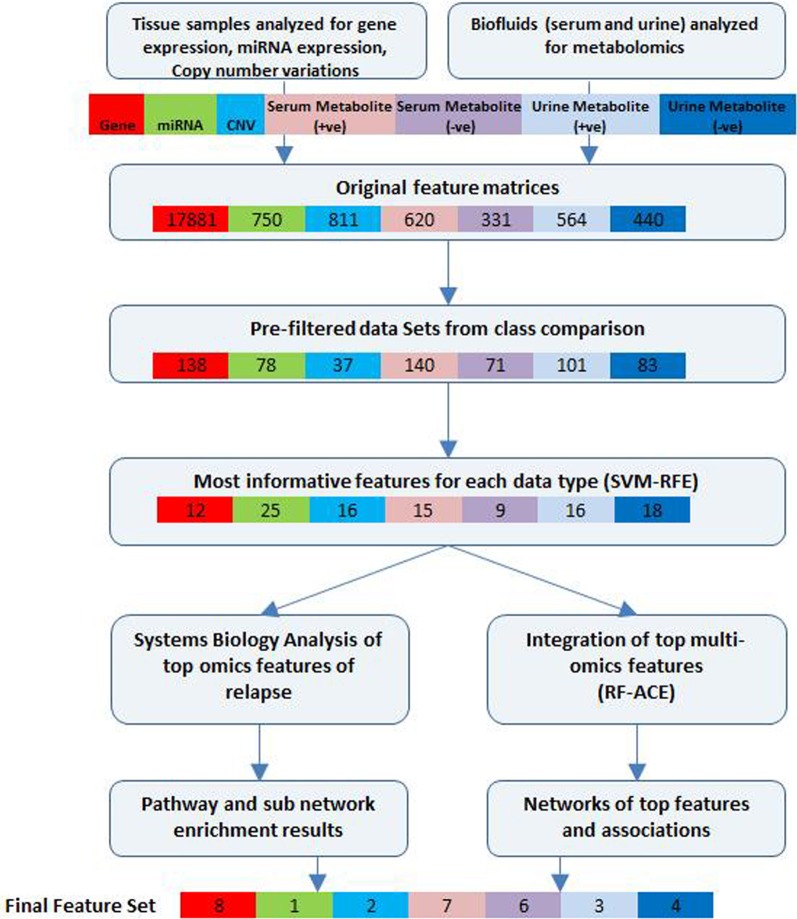
**Bioinformatics workflow of multivariate analysis**. Feature selection workflow for multi-omics profiling data from colorectal cancer patient samples (for relapse outcome) shown. Feature numbers for each data type are displayed [Gene—red, miRNA—green, CNV—cyan, serum metabolites (positive)—pink, serum metabolites (negative)—purple, urine metabolites (positive)—blue, urine metabolites (negative)—dark blue].

A schematic of our approach to identify the most informative multi-omics markers of CRC relapse is shown in Figure 1. We utilized frozen and paraffin tissue samples from 20 relapse and 20 relapse-free patients; the minimum follow up on each patient is five years post-surgery. The clinical properties of these 40 sample sets are described in Table 1. Samples were collected at the time of surgery prior to initiation of any treatment. The clinical analyses using KM plots and Cox regression analysis (Supplemental Figure 1) showed that tumor stage and other variables related to clinical chemistry parameters—Glucose, Bilirubin, Creatinine, CRP, and triglycerides may be putatively associated with relapse status and survival. Some of the confidence intervals of the hazard ratios are extremely large and indicate that they are not stable. Several clinical variables known to be linked with colon cancer such as use of alcohol, tumor grade, BMI and gender did not show significant association with outcome, indicating that the use of clinical data alone with low sample size does not give enough predictive power. Therefore, we did not do any further analysis with clinical data (no model adjustment was done), and focused our investigation on the genomic data to see if it had better predictive power.

**Table 1:** **Patient demographics, pathology, and biochemical characteristics**.

**Characteristics**	**No: (%)**
**GENDER**	
Male	22 (55)
Female	18 (45)
**TUMOR GRADE**	
Grade 2	33 (82.5)
Grade 3	7 (17.5)
**TUMOR STAGE**	
I	12 (30)
II^*^	24 (60)
IIA^*^	4 (10)
**RELAPSE/RECURRENCE**	
Yes	20 (50)
No	20 (50)
**VITAL STATUS**	
Alive	36 (90)
Dead	4 (10)
**AGE**	
≤40	1 (2.5)
41–55	3 (7.5)
56-70	25 (62.5)
>70	11 (27.5)
**TOTAL NUMBER OF LYMPH NODES**	
10-20	19 (47.5)
20-30	9 (22.5)
30-40	6 (15)
40-50	3 (7.5)
50-60	3 (7.5)
**GLUCOSE**	
<60 mg/dl	0 (0)*s*
60–100 mg/dl (reference range)	21 (52.5)
>100 mg/dl	10 (25)
Unknown	9 (22.5)
**BILIRUBIN**	
≤1.1 mg/dl (reference range)	36 (90)
>1.1 mg/dl	1 (2.5)
Unknown	3 (7.5)
**CRP LEVEL, C-REACTIVE PROTEIN)**	
≤5 mg/l (reference range)	25 (62.5)
>5 mg/l	11 (27.5)
Unknown	4 (10)
**CREATININE**	
≤1.1 mg/dl (reference range)	32 (80)
>1.1 mg/dl	4 (10)
Unknown	4 (10)
**BMI (BODY MASS INDEX)**	
Underweight, BMI <18.5)	1 (2.5)
Normal, BMI ≥18.5 and < 25)	17 (42.5)
Overweight, BMI ≥25 and < 30)	12 (30)
Obese, BMI ≥30)	10 (25)
**DISEASE LOCALIZATION**	
Sigmoid colon	14 (35)
Ileocaecal	4 (10)
Left flexure	1 (2.5)
Rectum	14 (35)
Transverse colon	2 (5)
Ascending colon	5 (12.5)

* Stage II refers to a tumor that has infiltrated into but not penetrated through the muscularis propria. Stage IIA is a “group staging” of tumor that includes nodal and metastatic status and indicates a tumor that has infiltrated into the outer layers of the colon (T3) but did not yet involve nodes (N0) and did not metastasize (M0) to distant organs.

Initial supervised analysis of tissue samples resulted in 138 gene expression features, 78 miRNAs, and 37 cytobands significantly associated with CRC relapse. Biofluid analysis resulted in 140 serum (+ve ESI mode) metabolites, 71 serum (−ve ESI mode) metabolites, 101 urine (+ve mode) metabolites and 83 urine (−ve mode) metabolites. Using a rigorous cross-validation approach of support vector machine learning algorithms and recursive feature elimination (SVM-RFE) combined with random forest based integrative analysis (RF-ACE) we reduced the most informative feature list to 8 genes, 1 microRNA, 2 cytobands, and 13 metabolites from serum and 7 metabolites from urine. We report on the details of the results below. Near 100% accuracy in classification was achieved for 12 genes and 13 serum metabolites (7 +ve mode and 6 −ve mode).

### Tumor tissue molecular profiling

#### A 12-gene panel predicts relapse

**Figure 2:**
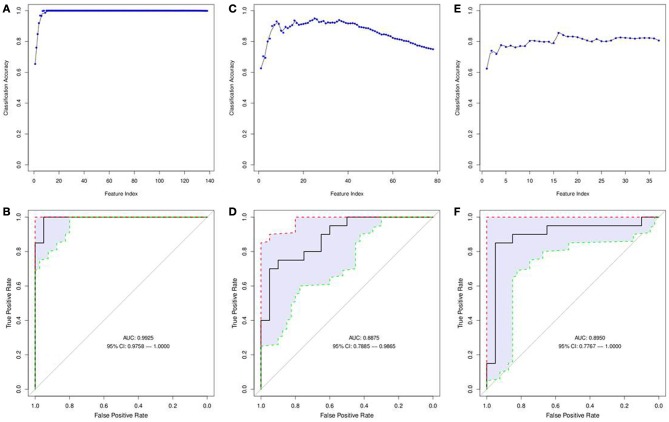
**Features selected by SVM-RFE machine learning method for tissue based analysis**. Minimal number of features were selected for each data type providing maximum accuracy of classification for clinical outcome. Gene expression SVM-RFE results **(A)** and ROC Curve showing confidence intervals **(B)** based on top 12 genes are shown. microRNA expression SVM-RFE results **(C)** and ROC Curve showing confidence intervals **(D)** based on top 25 microRNAs are shown. DNA copy number alterations by CIN Index for cytobands are shown. SVM-RFE **(E)** and ROC Curve showing confidence intervals **(F)** based on top 16 cytobands.

Normalized gene expression data were filtered by significance using a *t*-test and further analyzed utilizing SVM-RFE to determine the most informative genes providing the best classification of relapse vs. relapse-free samples (Figures 2A,B). A total of 12 genes were identified that provide maximum accuracy of classification (Table 2). The results of SVM-RFE analysis were computationally validated using a leave-one out approach and resulted in near 100% accurate classification (95% confidence interval: 0.9758–1.000) of the samples in two groups—relapse vs. relapse-free.

**Table 2:** **Short list of 12 genes from SVM**.

**ID**	**Entrez gene name**	**Location**	**Type(s)**
ARHGAP28	Rho GTPase activating protein 28	Cytoplasm	Other
CDA	Cytidine deaminase	Nucleus	Enzyme
CEACAM19	Carcinoembryonic antigen-related cell adhesion molecule 19	Unknown	Other
CXCL11	Chemokine (C-X-C motif) ligand 11	Unknown	Cytokine
CXCL13	Chemokine (C-X-C motif) ligand 13	Extracellular space	Cytokine
DNASE1L1	Deoxyribonuclease I-like 1	Cytoplasm	Enzyme
IFIT5	Interferon-induced protein with tetratricopeptide repeats 5	Plasma membrane	other
IRAK3	Interleukin-1 receptor-associated kinase 3	Cytoplasm	KINASE
OR6S1	Olfactory receptor, family 6, subfamily S, member 1	Plasma membrane	G-protein coupled receptor
PCOLCE2	Procollagen C-endopeptidase enhancer 2	Extracellular space	Other
PDE9A	Phosphodiesterase 9A	Cytoplasm	Enzyme
TNIP3	TNFAIP3 interacting protein 3	Unknown	Other

#### Pathways and biological processes involved in CRC relapse

**Figure 3:**
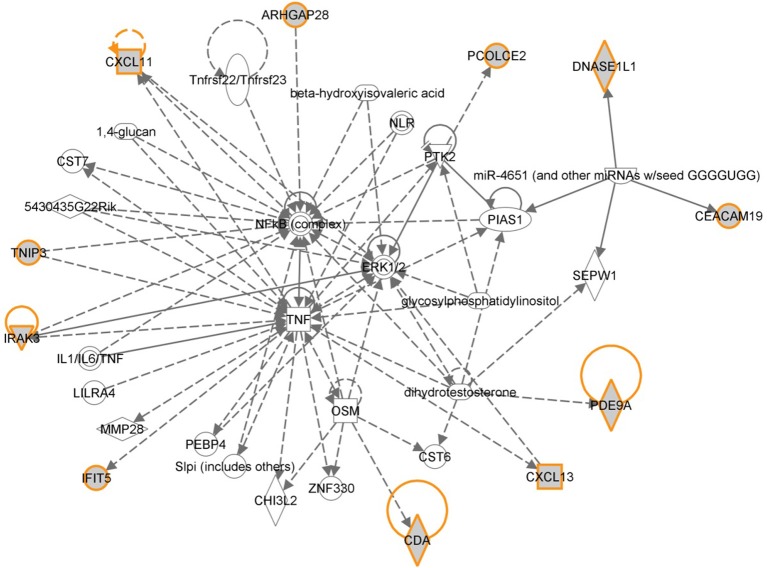
**Gene network shown for the short list of selected genes in Table 2**. Genes in the short list are shown with yellow outline. Direct downstream interactions include TNF, NFkB, and ERK1/2.

The 12 most informative genes analyzed for pathway enrichment and GO categories identified biological processes and pathways related to immune response, immune cell signaling, and trafficking (Supplemental Table 1). Eleven out of twelve genes (the exception being OR6S1) were found to be a part of known interaction networks (Figure 3) with major biological functions involving cell mediated immune response, cell movement and hematological system development and function. For example immune responders such as chemokines (e.g., CXCL11) and cytokine signal transducers including interleukin-1 receptor associated kinase (IRAK)-M, also known as IRAK3 were down-regulated in the relapse cases. This finding supports the results of our pathway analysis of 138 differentially expressed genes where we also found significant enrichment of immune response categories. The fact that after recursive feature elimination 11 of the 12 most informative genes are directly involved in immunological functions underlines a central role for immune response alterations in clinical outcome of relapse.

#### MicroRNAs (miRNAs) involved in immune response regulation detected

Normalized gene expression data were filtered by significance using *t*-test and further analyzed utilizing SVM-RFE to determine the most informative miRNAs providing the best classification of relapse vs. relapse-free samples (Figures 2C,D). Out of 80 differentially expressed microRNAs, 25 provided maximum accuracy of classification (Supplemental Table 2). The results of SVM-RFE analysis were computationally validated using a leave-one out approach and resulted in 88% accuracy in classification (95% confidence interval: 0.7885–0.9855) of relapse and relapse-free samples.

We conducted downstream systems biology analysis of targets of the top 25 microRNAs from SVM-RFE using combinatorial target enrichment analysis for KEGG pathways (miRPath v.2.0 (Vlachos et al., 2012) and gene ontology enrichment analysis tools from the MiRo software package (Giskeodegard et al., 2010). The CRC pathway was significantly enriched with 26 targets of 19 microRNAs from the top 25. In addition, pathways relevant to immune response signaling (Supplemental Table 3), T-cell receptor signaling (39 genes by 20 microRNAs), B-cells receptor signaling (34 genes by 20 microRNAs) and chemokine signaling (70 genes by 20 microRNAs) were also enriched (Supplemental Figure 2). One of the highly-ranked microRNAs, miR-934, is predicted to target APC as well as multiple target genes within categories such as antigen presentation (AP3B1, HLA-DPB1), immune response (HLA-DPB1, LILRB4, FYB, IL1F5, CLEC5A, CRTAM, CTSS, CCL7, CD300LF, IL20) and inflammatory response (THBS1, F11R, CCL7, ATRN, IL1F, CLEC7A, C6). In a consecutive multivariate analysis by RF-ACE, microRNA-934 was found to be the top ranked microRNA significantly associated with relapse in our analyses (importance score: 0.0189).

Overall, pathway and gene ontology enrichment analysis of microRNA targets for the 25 top microRNAs indicated involvement of these microRNAs in the regulation of many genes in pathways related to T and B cell signaling and regulation of immune response by chemokines.

#### DNA copy number alterations

Data on DNA copy number analysis were used to calculate the chromosome instability (CIN) index at the whole chromosome and cytoband levels. CIN index data were filtered by significance using a *t*-test and further analyzed utilizing SVM-RFE to determine the most informative panel of cytobands that provided the best classification of relapse vs. relapse-free samples (Figures 2E,F). Sixteen cytobands were identified that provided maximum accuracy of classification (Supplemental Table 2). The results of SVM-RFE analysis were validated using a leave-one-out cross-validation approach and resulted in 95% accuracy in classification (95% confidence interval: 0.7767–1.000) of the relapse and relapse-free samples.

**Figure 4:**
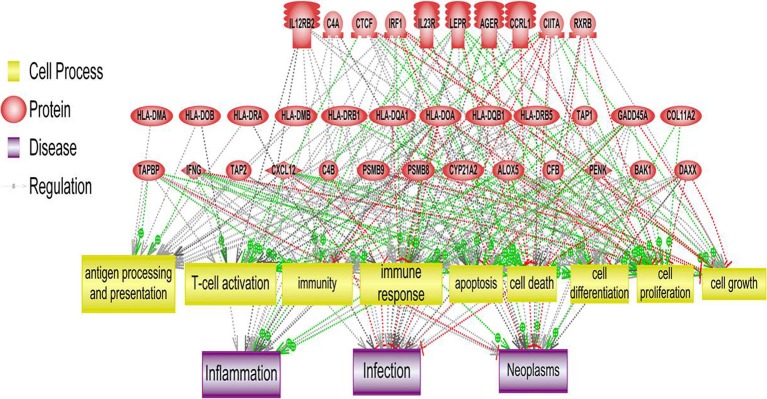
**Systems biology analysis of amplified and deleted cytoband regions**. Downstream analysis of genes located on cytobands with significantly abnormal CIN index in cohort of patients with relapse shown. Biological processes affected the most by genes located on significantly altered cytobands include antigen processing and presentation, T-cell activation and immune response.

As reported earlier (Brosens et al., 2010), we observed 4q deletions in the relapse cases. A systems biology analysis was performed to identify the functional role of genes located on those cytobands with significant CIN index indicating genomic instability. This analysis determined several biological processes and pathways related to immune response, immune cell signaling and trafficking, as well as cancer, cell cycle regulation, and cell proliferation (Figure 4).

Additional analysis was done at the level of gains and losses related to CIN index; genes located on cytobands with significant loss or gains were further analyzed separately. Pathway enrichment of the genes located on cytobands with gains resulted in 11 Gene Ontology cell processes related to either immunity or inflammation. These processes included T cell receptor signaling, T cell co-stimulation, positive regulation of T cell mediated cytotoxicity, and cytokine-mediated signaling. The least statistically significant pathway had a *p*-value of 0.0035.

Pathway enrichment of genes from cytobands with loss resulted in three gene ontology based cell processes related to either immunity or inflammation: (1) positive regulation of interleukin-17 production (*p* < 0.001), (2) negative regulation of activated T cell proliferation (*p* = 0.0026); and (3) positive regulation of natural killer cell differentiation (*p* = 0.0026). In addition, several other GO categories related to cancer, cell cycle and cell proliferation were enriched.

#### Mutation analysis

**Figure 5:**
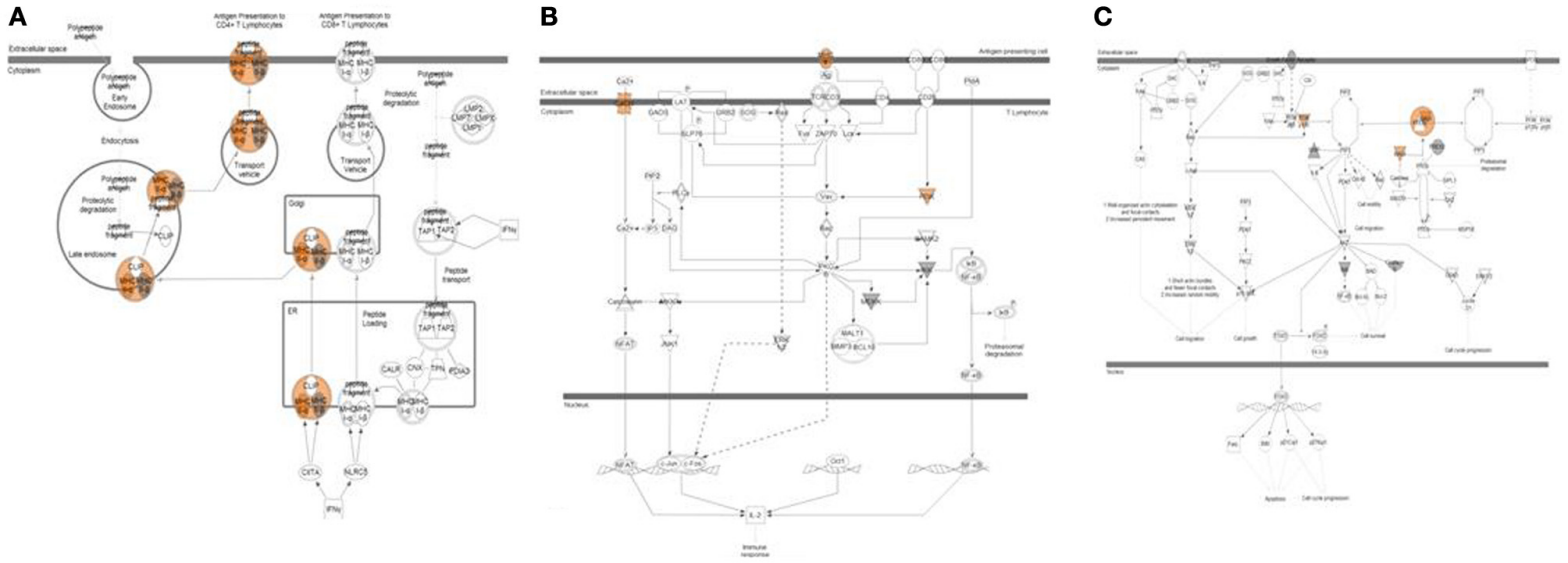
**Pathway analysis of top variants from exome sequencing data**. Three pathways highly enriched with non-synonymous variants found exclusively in relapse patients are shown. **(A)** Antigen presentation pathway affected in 8 relapse cases shown. Variants with predicted activation effect were mapped predominantly to one branch of pathway related to activation of CD4+ Lymphocytes. **(B)** PKC-Theta Signaling in T-Lymphocytes affected in 14 relapse cases shown. **(C)** PTEN Signaling affected in 14 relapse cases shown.

Data from exome sequencing analysis were processed to identify mutations in the tumor samples. Variants were annotated and filtered to determine a subset of mutations that are most likely to affect protein structure and/or function in samples with relapse and were not present in relapse-free samples (Supplemental Figure 3A). Several distinct types of variants were detected including variants in gene coding regions, 3′-UTRs, and in non-coding RNA genes (Supplemental Figure 3B). A full list of filtered, non-synonymous variants is shown in Supplemental Table 4. Systems biology analysis of pathways and biological processes allowed us to map these subsets of variants to specific pathways that are enriched with mutations found in our analysis. Several categories relevant to known cancer related pathways were found as well as biological processes related to T-cell activation and antigen presentation (Table 3; Figure 5). Variants in 8 relapse cases were mapped predominantly to one branch of the antigen presentation pathway related to activation of CD4+ Lymphocytes. Variants in genes involved in PKC, PKC-Theta, and PTEN Signaling pathways were found in 14 of the relapse cases and in none of the relapse-free cases.

**Table 3:** **Pathway enrichment for variants present only in relapse cases**.

**Name**	***p*-Value**	**No. of genes**	**No. of variants**	**No. of cases**	**No. of controls**
PKC_Theta, signaling in T lymphocytes	3.98E-02	14	17	14	0
PTEN signaling	2.68E-02	12	12	14	0
iCOS-iCOSL signaling in T helper cells	4.24E-02	11	13	11	0
Amyotrophic lateral sclerosis signaling	6.56E-03	12	12	11	0
Altered T cell and B cell signaling in rheumatoid arthritis	3.39E-02	10	12	11	0
CTLA4 signaling in cytotoxic T lymphocytes	1.01E-02	11	12	10	0
IL-4 signaling	1.54E-03	10	12	9	0
Calcium-induced T lymphocyte apoptosis	4.05E-02	8	10	8	0
Nur77 signaling in T lymphocytes	3.18E-02	8	9	8	0
Graft-versus-host disease signaling	4.04E-02	7	8	8	0
Antigen presentation pathway	6.03E-03	6	7	8	0
B cell development	3.17E-04	7	8	7	0
Complement system	1.24E-03	7	7	7	0
Role of BRCA1 in DNA damage response	1.64E-02	7	7	7	0
IL-17 signaling	3.83E-02	7	7	7	0
Regulation of IL-2 expression in activated and anergic T lymphocytes	2.68E-02	1	1	6	0
Phospholipase C signaling	2.77E-02	6	7	5	0
Signaling by rho family GTPases	1.21E-02	4	4	4	0
Tec kinase signaling	1.38E-02	3	3	3	0
Leukocyte extravasation signaling	2.88E-02	3	3	3	0
RhoGDI signaling	6.63E-03	2	2	1	0

Total of 21 pathways are significantly enriched. Table is sorted by number of cases showing these variants within genes in the enriched pathways.

Burden testing (Li and Leal, 2008) was performed on variant data obtained from exome sequencing with a focus on rare variant detection to ensure that the presence of more common mutations does not affect major trends detected by Ingenuity® Variant Analysis (IVA). Results from burden testing were comparable to the analysis in IVA. Enrichment analysis of the burden test of genes from tumor samples (relapse vs. relapse-free) resulted in several immune-related and inflammatory pathways, including: innate immune response (*p* = 0.0024); neutrophil degranulation (*p* = 0.0052); inflammatory response (*p* = 0.0056); negative regulation of interferon-alpha biosynthetic process (*p* = 0.016); positive regulation of chemokine (C-C motif) ligand 5 production (*p* = 0.016); interferon-gamma-mediated signaling pathway (*p* = 0.035); and positive regulation of interleukin-8 production (*p* = 0.037).

In summary, variant data point to biological processes and pathways related to immune system response such as T- and B-cell activation and antigen presentation as being affected in patients destined to relapse when compared to those destined to be relapse-free.

### Biofluid profiling results

Metabolomic profiling data were generated from serum and urine samples collected immediately prior to surgery from the same cohort of patients used for tissue profiling results.

#### Serum metabolomics profiles

**Figure 6:**
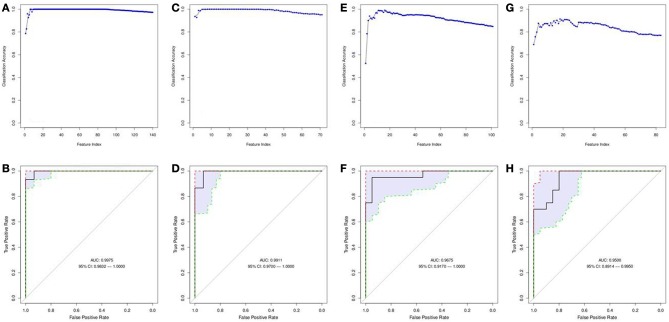
**Features selected by SVM-RFE machine learning method for biofluids based analysis**. Results of feature selection for metabolomics data in biofluids samples by SVM-RFE and ROC curves with confidence intervals are shown. Minimal number of features were selected for each data type providing maximum accuracy of classification for clinical outcome. Metabolites in Serum (positive charge) SVM-RFE results **(A)** and ROC Curve **(B)** based on top 15 metabolites. Metabolites in Serum (negative charge) SVM-RFE results **(C)** and ROC Curve based **(D)** on top 9 metabolites. Metabolites in Urine (positive charge) SVM-RFE results **(E)** and ROC Curve **(F)** based on top 16 metabolites. Metabolites in Urine (negative charge) SVM-RFE results **(G)** and ROC Curve **(H)** based on top 18 metabolites.

A matrix of m/z values for features from serum samples (positive and negative charge) was used to filter for significantly different metabolites between relapse and relapse-free groups and further analyzed utilizing the SVM-RFE algorithm to determine the metabolites that provide the best classification of relapse vs. relapse-free. Fifteen features comprised the serum positive dataset (Figures 6A,B) and 9 features for the serum negative data set (Figures 6C,D). Twenty-four serum features/metabolites provided maximum accuracy (near 100%) of classification with a 95% confidence interval of 0.9832–1.000 for the positive mode and a 95% confidence interval of 0.9700–1.000 for the negative mode (Supplemental Table 2).

#### Urine metabolomics profiles

Similarly, a matrix of m/z values for features from urine samples (positive and negative charge) was filtered on significance by *t*-test and then analyzed with SVM-RFE algorithms. Sixteen features from the urine positive dataset (Figures 6E,F) and 18 features from the urine negative dataset (Figures 6G,H) were identified. These 34 urine features/metabolites provided a maximum accuracy of classification (95%) with a 95% confidence interval of 0.9170–1.000 for the positive mode and a 95% confidence interval of 0.8914–0.9950 for the negative mode (Supplemental Table 2).

Leave-one-out cross validation was performed to ensure reproducibility of classification results for both serum and urine based metabolomics data. These two sets of metabolites were combined and annotated using an in-house metabolomics annotation pipeline (under review, *BMC Bioinformatics*, briefly described in Methods). A total of 25 putative metabolites from serum and 76 metabolites from urine were annotated and mapped to known pathways (Table 4). Since multiple candidate metabolites with similar m/z ratios were annotated by this pipeline, the list of putative metabolites was manually curated to select the most likely candidate for each m/z peak. The final list of 25 putative metabolites from urine and 6 metabolites from serum was added to a combined list of most informative features and further analyzed using multivariate analysis methodology (results below).

**Table 4:** **Pathway enrichment analysis. Enrichment of top most informative metabolites in serum and urine**.

**Pathway**	***p*-Value**	**Members_input_overlap**	**%**	**Source**
**URINE (POS+NEG)**				
L-dopachrome biosynthesis	0.000593058	HMDB01229; HMDB04067	2 (28.6)	HumanCyc
Caffeine metabolism	0.001835499	HMDB03099; HMDB11107	2 (16.7)	SMPDB
Caffeine metabolism—homo sapiens (human)	0.00417735	HMDB03099; HMDB11107	2 (11.1)	KEGG
Dopamine metabolism	0.008658165	HMDB01229; HMDB04067	2 (7.7)	Wikipathways
Tyrosine metabolism	0.01805096	HMDB01229; HMDB04067	2 (5.3)	SMPDB
Tyrosine metabolism—homo sapiens (human)	0.038659718	HMDB01229; HMDB04067	2 (3.5)	KEGG
**Pathway**	***p*-Value**	**Members_input_overlap**	**Candidates contained**	**Source**
**SERUM (POS+NEG)**				
Bile acid biosynthesis	0.001594724	HMDB00631; HMDB00637	2 (4.3)	SMPDB

Several annotated metabolites in serum and urine are involved in signaling and/or regulation of immune response and inflammation. Chenodeoxyglycocholic acid in serum has been reported as one of the metabolic biomarkers of Crohn's Disease (Jansson et al., 2009). Notoginsenosides were reported as immunologic adjuvants (Sun et al., 2006). 4-Hydroxy-2-butenoic acid gamma-lactone in the serum positive group is known to modify T and B cell mediated immune responses (Ritchie et al., 2003). Carnitine metabolites are altered in kidney cancers (Ganti et al., 2012) and are involved in immune and inflammatory responses.

### Combining molecular features from tissue and biofluids

Assuming that molecular profiling features of different types might provide complementary information with regard to association with clinical outcome we have applied multivariate analysis using a modified version of the Random Forest algorithm called RF-ACE (http://www.genome.gov/Multimedia/Slides/TCGA1/TCGA1_Erkkila.pdf) to find the best combination of tissue based and biofluid based molecular correlates of relapse. As a result, a combined list of multi-omics features were ranked based on importance score indicating the degree of association of each feature with future clinical relapse (the significance of association was determined by *p*-values). Additional information was generated with regard to mutual interconnection of various features based on Kendall rank correlation.

The resulting list of candidate biomarkers was filtered on a p-value threshold of 0.01 and was further analyzed using the Regulome Explorer network visualization tool. A list of features ranked by importance score is presented in Supplemental materials (Supplemental Table 5).

**Figure 7:**
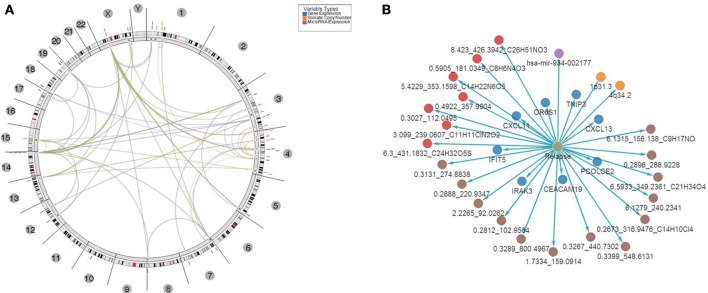
**Top 31 features associated with relapse. (A)** Results of multivariate analysis and integration of tissues-based and bio-fluid based top omics features. Features were mapped onto genomic coordinates on a circos plot, with edges indicating pairs of features with significant association of changes between them found in samples with relapse. Several clusters of features were evident with “hotspots” at chromosomes 1, 3, 4, and x. **(B)** Top multi-omics features associated with relapse ranked based on *p*-value of significance of association *P* ≤ 1E-30.

This set of multi-omics features was further analyzed and mapped to the relevant human genome location using Regulome Explorer circos plots and network representation of correlations between the features. Mapping based on the association among genes, microRNA, CNVs and other features yielded multiple “hubs” at various genomic coordinates (Figure 7A) with multiple features clustered at chromosomes 1, 3, 4, 14, and X. A small subset of features had direct significant association to relapse with *p* ≤ 1E-30 (Figure 7B; Supplemental Table 6) and consisted of 8 genes, 1 microRNA, 2 cytobands, 13 metabolites from serum, and 7 metabolites in urine. Among 8 genes from this list of top predictors of relapse, five were previously identified as directly involved in regulation of immune response, as well as microRNA- 934 that was annotated as targeting immune response, antigen presentation and inflammatory response. Cytoband 4q34.2 was among those cytobands that had a significant CIN index associated with copy number loss. Genes located on this cytoband were related to immune response and T- and B- cell trafficking as indicated previously (*DNA copy number alterations*).

In addition, network based mapping has shown a high degree of correlation between several feature types such as metabolites and several genes and microRNAs indicating a computationally based association of biofluid based markers with aberrantly expressed features in tumor tissue. Network representation of these associations has identified several “hubs” among the top genes that are relevant to the biological processes of immune response, antigen presentation and cytokine regulation of lymphocyte trafficking. For example a network of genes and correlated metabolites in urine (Supplemental Figure 4) revealed high connectivity with 4 genes—CXCL13, TNP3, IFIT5 and CDA indicating that biofluid derived metabolite analysis might be relevant to the same underlying biological processes that were detected in tissue i.e. regulation of T-cell activation and lymphocyte trafficking in the context of CRC clinical biology.

### Validation of molecular results by histopathological analysis of tumor sections

Previous studies have shown that lymphocyte infiltration of tumors provides a protective anti-tumor response (Deschoolmeester et al., 2010; Liu et al., 2012). To corroborate prior results and the results of our molecular profiling analyses, we performed a blind immunohistochemical assessment of tumor sections from a subset of 15 cases representing 7 relapse and 8 relapse-free patients. We hypothesized that the combination of molecular features we found to be associated with relapse could regulate T-cell and B-cell activation in patients leading to differences in tumor lymphocyte content.

**Figure 8:**
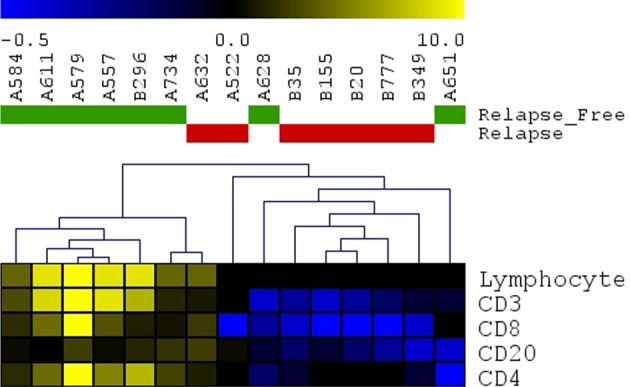
**Immunohistochemistry detection of infiltrating lymphocytes**. Scoring results for CD3, CD4, CD8, CD20 detection as well as overall scoring of infiltrating lymphocytes (lymphocyte component). Heatmap presenting unsupervised hierarchical clustering results for 15 cut sections (7 samples with relapse and 8 samples without relapse). Relapse samples clustered together except for sample#A632. Relapse-free samples clustered together except for 2 samples (A628 and A651).

**Figure 9:**
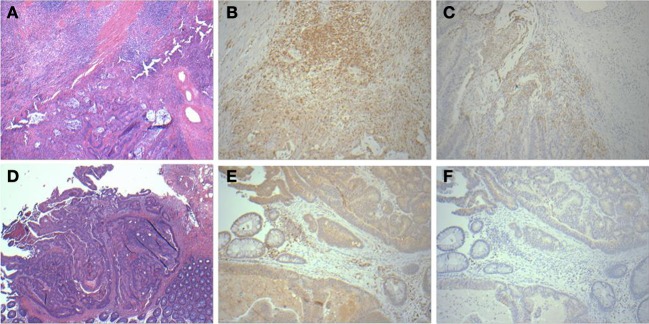
**Histopathological staining showing enhanced infiltration in relapse-free cases**. HandE **(A)**, CD3 **(B)** and CD8 **(C)** for sample A579 (relapse-free) showing prominent lymphoid component which is T-cell (CD3) and also showing CD8 positive cytotoxic T-cells surrounding the tumor. Corresponding pictures from B349 (relapse case) showing HandE staining **(D)** and markedly decreased lymphoid component with few CD3 positive T-cells **(E)** and almost no CD8 positive cytotoxic T-cells **(F)**.

A panel of antibodies recognizing CD3, CD4, CD8, and CD20 permitted the detection of the major T- and B-lymphocyte subsets. IHC staining results were scored in a blinded fashion for each marker and scores were compared between the relapse and relapse-free groups. The results of histological evaluation and scoring demonstrated a significantly higher fraction of infiltrating CD3 and CD8 lymphocytes in the relapse-free cases (Figure 8). Figure 9 shows a representative images with a high content of CD3 (Figure 9B) and CD8 (Figure 9C) staining in sample A579 (relapse-free) while IHC staining of B349 tumor (relapse) showed a markedly decreased lymphoid component with few CD3 positive T-cells (Figure 9E) and almost no CD8 T-cells (Figure 9F). We observed down-regulation of many cytokines related genes in relapse patients. This observation is consistent with an overall decrease in infiltrating lymphocytes as they are controlled and/or activated by cytokines. Detailed enrichment analysis of top 12 genes SVM cross-validation analysis showed biological functions relating to lymphocyte migration, chemotaxis and attraction of lymphocytes including specific subpopulations of Th0, Th1, B1 and memory lymphocytes (Supplemental Table 1). For example, CXCL11 and CXCL13 cytokine genes that are regulators of T- and B- lymphocytes were down-regulated in relapse cases. The downregulation of these genes in the relapse group correlates with a potential downregulation of related biological functions as detected by histopathology assessment of infiltrating cells in tumor cut sections. These findings are also in accord with previous studies by Galon et al (Pages et al., 2005; Galon et al., 2006).

## Discussion

Cancer is increasingly becoming a BIG DATA problem. While looking at a single data type (such as driver mutations) has served us well in a small percentage of patients with non-small cell lung cancer (Shaw et al., 2013) and in patients with chronic myeloid leukemia (Yeung and Hughes, 2012), scientists are beginning to question the core premises of leading models of cancer therapy wherein cells become malignant when they develop mutations leading to uncontrolled proliferation. A recent study on the development and progression of colon cancer demonstrated that DNA alone is not the sole driver of a tumor's behavior (Kreso et al., 2013). In this study we have shown in stage II and stage I CRC patients that serum and urine metabolomics signatures have a very high accuracy of prediction when compared to somatic mutations alone. A mounting body of evidence suggests the need for an integrated approach, combining information on cellular properties, metabolites, and post-translational modifications of proteins in addition to genomic and patient phenotype information to enhance provide better understanding of clinically relevant cancer biology (Ge et al., 2003; Toyoda and Wada, 2004; Joyce and Palsson, 2006).

We have integrated the results of molecular profiling of several omics data types to determine the most reliable prognostic molecular correlates for relapse in CRC. The top 31 features were identified that highly correlated with relapse and consisted of 8 genes, 1 microRNA, 2 cytobands, 13 metabolites from serum, and 7 metabolites in urine. The data types were integrated using multistep analytical approach with consecutive elimination of redundant molecular features. A computational analysis was performed based on SVM-RFE algorithm for each data type to determine the minimal number of most informative features allowing for the best classification accuracy of future relapse. For each data type a systems biology analysis was performed to identify pathways, biological processes and disease categories that are affected the most based on short lists of features determined by SVM-RFE. To further investigate the relative contributions of all data types a multivariate analysis was conducted on a combined matrix of the most informative features using a novel method that is an improvement over the standard random forest analysis of heterogeneous features. As a result, multi-omics features were ranked based on degree of association with the clinical outcome of relapse.

A system biology focused analysis of a panel of multi-omics candidate biomarkers revealed major biological pathways and processes that are affected by the molecular anomalies in patients with relapse when compared with relapse-free patients. The results of integration were further analyzed by mapping multi-omics features onto genomic locations using a circos plot provided by the tool Regulome Explorer. These integrative and systems biology analyses suggest the relevance of tumor-immune system interactions and cytokine regulation of immune response in affecting disease outcome. This was reflected in the molecular changes observed at the level of genes, microRNAs, DNA copy number variation, and single nucleotide variations.

### Inflammation in colorectal cancer

The role of immune cells and the inflammatory response has been established in several types of cancer. The presence of immune cells and inflammation has been documented in every stage of cancer—from tumorigenesis to metastasis (Grivennikov et al., 2010). In CRC, the functionality (i.e., Th1 vs. Th2 vs. T-reg vs. Th17), relative density, and location (relative to tumor tissue) of immune cells all influence clinical outcome, regardless of tumor staging (Tosolini et al., 2011). Previous studies have demonstrated the prognostic implications identifying tumor-specific immune cells via differential gene expression analyses and in situ immunohistochemistry. Similarly, the differential expression and presence of particular cytokines and chemokines can also influence tumor progression, and in some cases can even be used for prognoses (Wang et al., 2009).

### Multi-omic signature of CRC relapse

**Figure 10:**
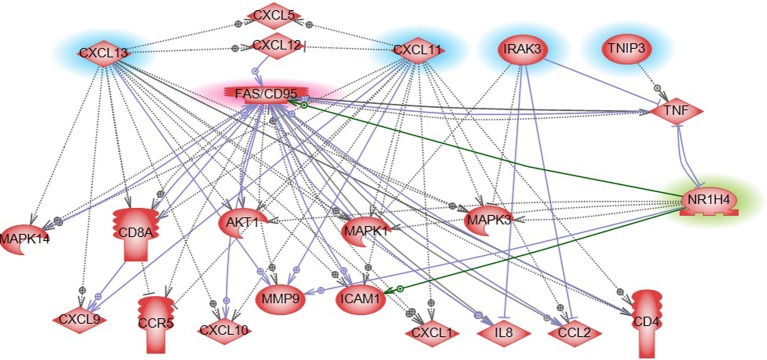
**Pathway analysis of FXR/NR1H4 gene**. Pathway analysis showed FXR in a complex network with significant genes identified from other omics data in our analysis. Blue highlight: Gene expression top features from SVM analysis; Red highlight: Key molecule in immune reaction in CRC (from literature); and Green highlight: Key molecule for serum metabolite bile acid- from literature.

Gene expression profiling provides a quick overview of gene activity and thereby the major events at the cellular level. A high proportion of the differentially expressed genes associated with CRC relapse phenotype were found to play a critical role in immune response functions. For example chemokines (CXCL11 and CLXL13) and cytokine signal transducers including IRAK3 were downregulated in relapse cases. CXCL11 has angiostatic properties and promotes the migration of cytotoxic T lymphocytes toward tumors triggering tumor cell apoptosis (Berencsi et al., 2007) while CXCL13, a B cell attracting chemokine is responsible for the development of secondary lymphoid tissue in the gut (Carlsen et al., 2002). IRAK3 is a negative regulator of the Toll-like receptor/Interleukin (IL)-1 receptor (TLR/IL-1R), which plays a fundamental role in the immune response (Janssens and Beyaert, 2003) and the NFKB pathway. TLR mediates the induction of pro-inflammatory cytokines and chemokines, We therefore performed a network analysis (Figure 10) of these and other genes from expression profiling data to understand a possible role for these genes in colon cancer recurrence. Interestingly, pathway analysis identified several common target genes, all of which had a complex interaction network with the TNF receptor, FAS (CD95) (Figure 10). CD95 is thought to play a crucial role in controlling colon tumor growth via tumor immune-surveillance. It is not only lost in a high percentage of CRCs (Moller et al., 1994), but is also impaired in patients who develop CRC relapse after curative-attempt surgery (Strater et al., 2005).

Integrative analysis of CNVs, gene expression and miRNA identified several regions with aberrant CIN index mapping to chr 1p, 3q, 4p, 4q, and 15q associated with CRC-relapse phenotype. Chromosomal instability especially of 4q (Brosens et al., 2011; Kodeda et al., 2012) and 15q (Brosens et al., 2011) have been previously associated with local recurrence in colon cancers after surgical resection. Pathway analysis of genes in these regions predominantly converged to immune response and inflammation processes, besides biological processes involving cell proliferation and cell-cycle progression (Figure 4).

A set of genes harboring potentially deleterious variants found in relapse sample was compared with most informative genes and microRNAs that were differentially expressed between relapse and relapse-free samples. No overlap was found indicating that most informative differentially expressed genes and microRNAs were not directly affected by deleterious mutations. However when upstream and downstream neighbors were considered, we found significant overlap of variant harboring genes with the genes upstream and downstream of DEGs. Similarly, we found overlap between genes harboring variants and microRNA target genes downstream of differentially expressed microRNAs. These finding indicate possible causative relationships between functionally significant mutations and aberrant expression of genes that are regulatory partners of mutated genes.

With respect to the metabolite profile, SVM-RFE analysis identified bile acid components as major metabolites in the CRC-relapse group. Bile acids, especially deoxycholate, in high cellular concentration promotes proliferation of colon cancer cells (Kawano et al., 2010) and are thought to play a major role in inflammation associated colon cancer (Wagner and Cohen, 1991; Modica et al., 2008; Gadaleta et al., 2010). Farnesoid X receptor (FXR/NR1H4) is a key regulator of bile acid metabolism (16037564) and its expression is often decreased or absent in CRC cells (Maran et al., 2009; Torres et al., 2013). Pathway analysis of FXR/NR1H4 gene showed FXR in a complex network with significant genes identified from other omics data in our analysis. Further, network analysis identified FXR as a critical component of nuclear receptors that regulates intestinal immunity via regulating the expression of cytokines, including TNFalpha (Figure 10).

Seven of the miRNAs identified in our analysis have been implicated in colon cancer based on published data from the Ingenuity knowledgebase, and for many, their impaired status has been reported in other cancers. Specific roles for these miRNAs have included cell proliferation, cellular senescence and tumor cell migrations (Ding et al., 2010; Kim et al., 2012; Li et al., 2012).

Enrichment analysis of microRNA targets for 25 microRNAs (Supplemental Table 2) showed that 26 of their predicted target genes mapped to the KEGG CRC pathway. These genes were targeted by at least one of the 19 microRNAs from the top 25. Several pathways related to T- and B-cell receptor signaling were also significantly enriched with multiple gene targets of these selected microRNAs (Supplemental Figure 2).

When top features from all data types were integrated using the RF-ACE method and filtered for high significance (*p* ≤ 1E-30), miRNA-934 was among the top 31 combined features selected. This miRNA has a computationally predicted gene target (APC) that plays a central role in CRC. The APC gene encodes a tumor suppressor protein involved in the WNT signaling pathway. Inappropriate activation of this pathway through loss of APC function has been shown to contribute to cancer progression in familial adenomatous polyposis (Rustgi, 2007). Several microRNA-934 target genes play a role in immune and inflammatory response, and antigen presentation (Lagana et al., 2009).

Finally, histological examination of frozen tissue sections from CRC patients with and without relapse was consistent with our findings of immune response genes as key predictors of CRC relapse (Figures 8, 9). The findings of tumor infiltrating CD8 and CD4 immune cells in the tissues from relapse-free patients is consistent with earlier reports on the reduction of CD8+ (Zlobec et al., 2008) and CD4+ cells (McMillan et al., 1997; Holcombe et al., 1999) as highly predictive of local recurrence of CRC while their presence associated with longer recurrence free survival (Holcombe et al., 1999; Chew et al., 2011; Muthuswamy et al., 2012).

These results suggest that a complex interaction between cancer cells and host immune mechanisms can predispose to either an anti- tumor or a pro-tumor environment. This interaction plays a critical role in not only tumor development and metastasis, but also tumor recurrence (Strater et al., 2005; de Souza and Bonorino, 2012). The present study was an attempt to identify molecular markers of CRC relapse from an integrative analysis of multi-omics data type, and the analysis consistently pointed to disruptions in genes involved in immune response and inflammatory processes associated with CRC relapse. We show that this integrated analysis model is feasible and could be utilized in informing decision making processes. Identification of involved pathways can also guide the selection of patients who may benefit from post-surgical chemotherapy with drugs that inhibit key genes in that pathway.

Metabolomics is a rapidly evolving field that aims to identify and quantify the concentration changes of all the metabolites in a given tissue or biofluid (i.e., the metabolome from a patient), usually in support of developing therapeutics or diagnostics. In fact, the anticipated contribution of metabolomics to the field of biomedicine is highlighted by its presence in the NIH Roadmap/NIH Common Fund initiatives. The application of metabolomics to help understand the manifestation(s) and progression of complex diseases like gastrointestinal (GI) cancers represents a powerful means to identify the earliest markers associated with phenotypic outcomes like recurrence and drug response. This method, if clinically validated can provide an economical and non-invasive method for prognostic and diagnostic purposes.

Projects such as TCGA provide comprehensive insights into functional anomalies relating to cell growth, proliferation, and immune response by comparing markers between normal and cancer tissue. This effort was aimed at cataloging changes at the molecular level in CRC relapse that can be detected years before the phenotypic changes surface by linking comprehensive multi-omic analyses to carefully defined clinical endpoints. It builds on prior knowledge from literature, public datasets, and experimental evidence to filter down to a few key players with potential for prognostication in CRC relapse. The immune response was the biologically most coherent signature that emerged from our analyses among several other biological processes, and corroborates other studies showing a strong immune response in patients less likely to relapse.

While promising, these discovery results are preliminary, and in most cases, validation of these potential immune biomarkers remains to be performed in appropriate future case-control validation trials. Nevertheless, there is an expectation that in the near future, some of these immune biomarkers will serve as reliable intermediate endpoints facilitating the management of patients with CRC and providing insight into the selection of the most effective therapeutic strategies for these patients.

## Materials and methods

### Patient cohort—clinical and demographic information

CRC patient biospecimens with extensive clinical and follow-up data were selected from the Indivumed GmbH biobank for 40 patients (20 relapse and 20 no relapse). The patients consisted of 12 with late stage I, and 28 with stage II (Table 1). Four patients (out of 12) with late stage I had experienced relapse (~33%), and it is important to note that 12 patients (out of 28) with stage II were relapse-free (~43%). Therefore, the relapse-free group of samples, and group with relapse are both represented by mixture of late stage I and stage II patients. Only nine stage II patients (out of 28) had rectal cancer; of these 6 had relapsed within 5 years.

A highly standardized process of biospecimen collection (e.g., documentation of time between surgical resection and postsurgical fixation and assurance of postsurgical fixation within 10 min) minimizes the risk of significant data variation because of pre-analytical factors such as fixation time after surgical resection. Of more than 180 clinical attributes, 64 were short listed based on relevance to clinical outcome and biomarker analysis. Key clinical characteristics are summarized in Table 1. Since the main clinical attribute of interest was “relapse,” it was important to understand which of the 64 attributes were relevant to relapse. For this, KM plots and Cox regression models were used to select the key clinical attributes (Supplemental Figure 1). Cox's proportional hazards model estimates relative risk and is widely used in the analysis of survival data to explain the effect of explanatory variables on survival times. Various subsets of clinical data were applied as input to the Cox model. Results from the Cox model as well as manual inspection of data elements by GI oncologists were selected to be important clinical attributes for correlation with molecular data.

### Sample preparation

All genomic analyses were performed on tumor tissue samples except for DNA copy number analysis, which included paired samples of tumor and adjacent non-tumor tissue. Adjacent non-tumor samples were used for normalization of copy number measurements.

#### RNA isolation and miRNA expression profiling

Total RNA, including miRNAs and other small molecules of RNA, were isolated from frozen tissue samples and extracted using the miRNeasy Mini Kit (QIAGEN, Valencia, CA), and from serum samples and extracted using the miRNeasy Serum/Plasma Kit (QIAGEN, Valencia, CA), according to the manufacturer's instructions. miRNA expression profiling was performed on 384-well format miRNA assays plates (Taqman Array Human MicoRNA A+B Cards, V3.0, Applied Biosystems, Foster City, CA) using qRT-PCR on a 7900HT Real-Time PCR System (Applied Biosystems, Foster City, CA).

#### RNA isolation and mRNA (Exon) expression profiling

Total RNA was isolated from frozen tissue samples and extracted using the RNeasy Mini Kit (QIAGEN, Valencia, CA) according to the manufacturer's instructions. Expression profiles were determined using Affymetrix GeneChip Human Exon 1.0 ST Arrays according to the manufacturer's instructions (Affymetrix, Santa Clara, CA, USA). The arrays were scanned using the Affymetrix GeneChip scanner 3000 7G system. Gene- and exon-level expression signal estimates were derived from cell intensity files (CEL) generated from Affymetrix GeneChip Exon 1.0 ST arrays.

#### DNA isolation and genome-wide SNP and CNV analysis

Genomic DNA was isolated from frozen tissue samples and extracted using standard salting out protocols which included proteinase K digestion followed by precipitation with phenol:chloroform:isoamyl alcohol (25:24:1). SNP and CNV data were obtained using the Affymetrix Genome-wide Human SNP array 6.0 according to the manufacturer's instructions (Affymetrix, Santa Clara, CA, USA). The arrays were scanned using the Affymetrix GeneChip scanner 3000 7G system with the Affymetrix Genotyping Console (version 4.1.2) software.

#### DNA isolation and exome sequencing

Genomic DNA was isolated as described above. Exome libraries were created according to the manufacturer's standard protocol for SOLiD library preparation (Applied Biosystems, Carlsbad, CA, USA). Three μg of genomic DNA was sheared via sonication using the Covaris (S-Series) instrument (Covaris, MA, USA). The ends of fragmented DNA were repaired and ligated to SOLiD P1 and A1 adapters provided in the Agilent Human All Exon 50 Mb Kit according to the manufacturer's instructions (Agilent, Santa Clara, CA, USA). The exomes were then captured using the Agilent Human All Exon 50 Mb Kit, and the amplified library was purified withAMPure XP beads (Beckman Coulter Genomics, Danvers, MA). Sequencing was performed using the Applied Biosystems SOLiD v4 sequencer (Life Technologies Corporation, CA, USA) using 50bp single end read libraries with 1 sample per quad (4 samples per slide).

#### Metabolomics profiling methods for biofluids

***Metabolite extraction.*** Urine samples were processed as described previously (Galon et al., 2006). Briefly, the samples were thawed on ice and vortexed. For metabolite extraction, 20 μL of urine was mixed with 80 μL of 50% acetonitrile (in water) containing internal standards [10 μL of debrisoquine (1 mg/mL) and 50 μL of 4-nitrobenzoic acid (1 mg/mL). For metabolite extraction from serum175 μL of 66% acetonitrile (in water) containing internal standards was added to 25 μL of plasma. The samples were incubated on ice for 15 min and centrifuged at 14,000 rpm at 4°C for 20 min. The supernatant was transferred to a fresh tube and dried under vacuum. The dried samples were resuspended in 100 μL of solvent A (98% water and 2% acetonitrile) for UPLC-ESI-Q-TOF-MS analysis.

***UPLC-ESI-QTOF-MS based data acquisition.*** Each sample (5 μL) was injected onto a reverse-phase 50 x 2.1 mm BEH 1.7 mm C18 column using an Acquity UPLC system (Waters Corporation, USA). The gradient mobile phase comprised of water containing 0.1% formic acid solution (A) and acetonitrile containing 0.1% formic acid solution (B). Each sample was resolved for 10 min at a flow rate of 0.5 mL/min.

The UPLC gradient consisted of 100% A for 0.5 min then a ramp of curve 6 to 60% B from 0.5 to 4.5 min, then a ramp of curve 6 to 100% B from 4.5 to 8.0 min, a hold at 100% B up to 9.0 min, then a ramp of curve 6 to 100% A from 9.0 to 9.2 min, followed by a hold at 100% A up to 10 min. The column eluent was introduced directly into the mass spectrometer by electrospray. Mass spectrometry was performed on a quadrupole-time-of-flight mass spectrometer operating in either negative or positive electrospray ionization mode with a capillary voltage of 3.2 kV and a sampling cone voltage of 35 V. The desolvation gas flow was 800 L/h and the temperature was set to 350°C. The cone gas flow was 50 L/h, and the source temperature was 150°C. The data were acquired in the V mode with a scan time of 0.3 s, and inter-scan delay at 0.08 s. Accurate mass was maintained by infusing sulfadimethoxine (311.0814 m/z) in 50% aqueous acetonitrile (250 pg/mL) at a rate of 30 mL/min via the lockspray interface every 10 s. Data were acquired in centroid mode from 50 to 850 m/z mass range for TOF-MS scanning for each sample in positive and negative ionization mode and checked for chromatographic reproducibility.

### Bioinformatics software platform

The primary platform for data analysis and integration for this study was G-DOC® (Georgetown Database of Cancer). The datasets from this study were loaded to G-DOC for further mining and analysis using the methods described (Madhavan et al., 2011). The G-DOC web portal (http://gdoc.georgetown.edu) includes a broad collection of bioinformatics and systems biology tools for analysis and visualization of four major “omics” types: DNA, mRNA, microRNA, and metabolites. By providing a powerful but easy to use interface, G-DOC was designed specifically to address the activation barrier for use of biomedical informatics tools by basic, clinical, and translational researchers. G-DOC contains a wide variety of analytic tools and capabilities, including integrated viewers for genomic features and three-dimensional drug-target complex structures. To help support effective patient group comparisons, G-DOC supports flexible clinical criteria browsing to enable selection of specific patient cohorts, and facilitates the generation of detailed reports and informative publication-quality plots. G-DOC also allows researchers to securely share knowledge with others through a powerful suite of collaboration-enabling features operating within its secure environment. This study is publicly accessible through the G-DOC web portal.

### Data processing

#### mRNA expression data

mRNA expression data processing was done as previously described (Madhavan et al., 2011). Briefly, pre-processing of microarray data primarily involves normalization with either RMA (Robust Multichip Average) (Irizarry et al., 2003) or Quantile Normalization (Bolstad et al., 2003) followed by log transformation of the data. More information on these standard normalization strategies is available at http://www.bioconductor.org. Significant post-processing effort is expended to ensure data quality and retention of the biological information provided.

#### miRNA expression data

RT-qPCR data were proccessed using comparative C(T) method (Livak and Schmittgen, 2001) and normalized to the average signal of endogenous controls (Schmittgen et al., 2008). These microRNA reporter Ids are mapped to mature miRNA accession numbers in miRBase (Kozomara and Griffiths-Jones, 2011) and hyperlinked to on-line public databases (miRBase, Entrez and iHOP), providing instant access to comprehensive microRNA genomic and deep sequencing information as well as predicted targets. miRNAs are also mapped on the genome using the JBrowse genome browser interface in G-DOC for integrative data visualization.

#### Metabolomics data

The metabolomics data for this study were processed into a data matrix format with samples as columns and features/metabolites as rows, and were normalized row-wise or column-wise in a sequential manner to minimize systematic variance and improve the performance for downstream statistical analysis. To annotate the metabolites, we used a home-grown annotation database and a knowledge driven network methodology (under review, BMC Bioinformatics). Briefly, we use a translational research workflow that allows integrative analysis of metabolomics data with other complementary ‘omics’ technologies including transcriptomics, proteomics and genomics using knowledge-driven networks. This network-based view of interconnected functional partners aids in bringing new insights about their mutual involvement associated with the phenotype of interest and more granular understanding of interdependence and interconnectivity between different underlying biochemical processes and pathways at a systems level. In conjunction, we use a fully cross-referenced database (MetPlus DB) by integrating the data from the three most comprehensive metabolite databases tailored largely toward mammalian metabolomics including HMDB, HUAMNCYC & LIPID MAPS with cross-referencing information for linking to several other mainstream chemoinformatics/bioinformatics repositories including KEGG, METLIN, ChEBI, FooDB, Pubchem, and Chemspider to provide unambiguous knowledge on clinically and physiologically relevant metabolites.

#### DNA copy number data

Raw data from Affymetrix SNPchip was pre-processed using D-Chip (Wong and Li, 2003) to extract a signal for individual probes. Piecewise constant segments of copy number profiles were estimated based on the Fused Margin Regression (FMR) method (Feng et al., 2010). Probe-level data were further proccessed to calculate copy number segments and chromosomal instability index (Kuo et al., 2009), one of the value-added analyses that come pre-generated within G-DOC. Segment data were used for calculation of CIN index at the level of whole chromosomes and individual cytobands (Kuo et al., 2009).

#### Somatic variant analysis

Whole exome data were pre-processed by vendor (EdgeBio) to the level 2 (TCGA, 2012) and BAM files were further analyzed using the Ingenuity Variant Analysis platform. A multi-sample VCF files was created for all 40 samples and uploaded to the private IVA cloud where the variant list was filtered to obtain a short list of non-synonymous, potentially deleterious variants. These variants were mapped to genomic regions, and further aggregated at the levels of gene, pathways, biological processes and diseases. The results of variant analysis are made available on-line as supplement material for the paper.

Additional statistical analysis was performed on variant data obtained from exome sequencing with a focus on rare variant detection to ensure that presence of more common mutations does not affect major trend detected by IVA analysis. Multi-sample VCF files for tumor samples were both analyzed using gene-level Burden test using the PLINK software package.

Genes that were determined by Burden tests as significantly affected by CNV in relapse group vs. relapse-free were further analyzed using pathway enrichment analysis to determine major biological processes that are significantly affected by detected variants.

### Statistical and bioinformatics analysis

#### Initial filtering

Normalized data were filtered on significance of changes between two groups of samples—with or without relapse using two-sided student *T*-test assuming unequal variance with p-values threshold at 0.05 and 0.01. A standard R-based package for *T*-test was used (Gentleman et al., 2004).

#### Individual data type analysis to identify best features

All results reported for individual data types were done using univariate analysis. Normalized and pre-filtered on significance data were analyzed using R-based package for Support Vector Machine (Bioconductor) with Recursive Feature Elimination (SVM-RFE). From the pre-filtered feature sets, we determined the most informative feature associated with the clinical outcome (relapse). All features for each data type were ranked by using the following iterative steps—(1) train the classifier using SVM; (2) compute the ranking criterion for all features; and (3) remove the feature with the smallest ranking criterion. Starting with the whole feature pool, we trained the SVM classifier based on the clinical information, and calculated the classification accuracy with leave-one-out cross-validations. Then the feature with minimum absolute weight (which is viewed as the feature contributing the least to the classification) was deleted from the classifier feature set. This cycle of calculations was repeated for each remaining feature until none were left. Using this recursive procedure, a subset of features was determined based on criteria for best performance of trained classifier with the minimal number of top ranked features.

#### ROC curves analysis

For each data type, a minimal number of features that provide maximum accuracy of classification were used to generate Receiver-Operator Curves (ROC). ROC curves were generated with 95% confidence intervals using the R package pROC (Robin et al., 2011), an open-source package to analyze ROC curves. Leave-one-out cross validation was used to validate the results of the ROC analysis and bootstrapping option was selected to generate confidence intervals.

#### Multivariate integrative analysis to rank heterogeneous features

The combined matrix of most informative features from each data type was generated by multivariate analysis using Random Forest with Artificial Contrast Elimination (RF-ACE). The RF-ACE overcomes potential issues identified in using just the Random Forest method, namely—the importance score yields mere ranking of associations, the importance score is not normalized, the prediction performance could be better, and existing RF implementations often lack flexibility. The RF-ACE implementation used in this study adds flexibility and improves performance over the standard Random Forest method. Features of this method include: support for string literals and a variety of data formats, normalized importance scores, inclusion of a statistical testing framework for associations, and better predictive power with Gradient Boosting Trees (http://www.genome.gov/Multimedia/Slides/TCGA1/TCGA1_Erkkila.pdf).

#### Addressing overfitting

Overfitting is a major problem when global profiling data are used to classify the samples. In our study, a multi-step data reduction, feature ranking, and various cross-validation procedures were applied to each type of omics data as well as during integration of multiple data types (Figure 1). In our analysis we have attempted to address this problem in several ways:

First, we pre-filtered data on significance of differences between case and control that led to a reduction in total number of features considered. Second, we applied the Recursive Feature Elimination algorithm in conjunction with SVM for each data type, which allowed ranking the features and selecting a minimal number of features allowing for maximum classification accuracy. The SVM-RFE algorithm has been reported in the literature as one of the best classification algorithms for addressing an overfitting for gene expression analysis (Guyon et al., 2002). For each data type this algorithm was applied with a rigorous cross validation procedure. At each step in SVM-RFE we use 2-fold cross-validation with 10,000 permutations. This was a variation of k-fold cross-validation. For each fold, we randomly assigned data points to two sets d0 and d1 (which were implemented by shuffling the data array and then splitting it in two), we then train on d0 and test on d1, followed by training on d1 and testing on d0. This has the advantage that our training and test sets are both large compared to the k-fold cross-validation method, and each data point is used for both training and validation on each fold (Picard and Cook, 1984; Arlot and Celisse, 2010). After this step, the number of features for each data type was reduced from hundreds to fewer than 30. Third, the ROC was calculated for each set of minimal number of features and validated using the leave-one-out cross-validation procedure. Forth, during integrative analysis we applied RF-ACE, which provided additional feature ranking based on importance score; this step involved the application of cross-validation with 10,000 random permutations. Although the total number of features was reduced to only 112 even before application of RF-ACE, this additional ranking procedure allowed us to narrow down the list of potential biomarkers to only 31.

Overall, the problem of overfitting was directly addressed in our analysis by multiple computational procedures of feature reduction, ranking, elimination and cross–validation, which were applied consecutively for individual data types as well as for combination of multiple molecular features. While we firmly believe that we have comprehensively addressed this computational problem known as overfitting in machine learning classification, a related biological issue of validation of classification results remains an open question and could only be addressed through additional experimental studies with a larger sample size of independently derived samples.

#### Integrative data visualization

The results of RF-ACE analysis were visualized using Regulome Explorer on-line tools (http://explorer.cancerregulome.org/). All data types were mapped to a circos plot with genomic coordinates. Correlation of features was represented as edges between corresponding nodes.

#### Systems biology analysis

Pathways and GO enrichment analysis for individual data types were performed using open source pathway enrichment analysis (Reactome) and commercial packages Ingenuity pathway analysis and Pathway Studio. Integrative network analysis of tissue based data types was done using subnetwork enrichment analysis (Pathway Studio) as well as commercial and open source tools for microRNA target analysis (miRPath 2.0, MiRo, TarBase 6.0, mirBase 18.0, and IPA microRNA analysis tools).

## Public access to data

G-DOC: https://gdoc.georgetown.edu/ (once you register, select CRC_MADHAVAN_2013_01)

**Exome sequencing data variant analysis:** https://variants.ingenuity.com/lvCRC_Georgetown_2013(Ingenuity requires free registration to access public datasets).

## Author contributions

Subha Madhavan, Yuriy Gusev, Stephen W. Byers, Hartmut Juhl, John L. Marshall, and Louis M. Weiner designed the study; Bhaskar Kallakury processed pathologic specimens; Lei Song, Krithika Bhuvaneshwar, Robinder Gauba, Yuriy Gusev, Abhishek Pandey, and Subha Madhavan conducted the computational and bioinformatics analysis; Bassem R. Haddad, David Goerlitz, and Amrita K. Cheema generated the data from high-throughput omics platforms; Thanemozhi G. Natarajan conducted literature search and drafted parts of the manuscript; Subha Madhavan, Yuriy Gusev, Stephen W. Byers, and Louis M. Weiner drafted the manuscript. All authors reviewed and edited the manuscript.

### Conflict of interest statement

The authors declare that the research was conducted in the absence of any commercial or financial relationships that could be construed as a potential conflict of interest.
