# Supplementary material for: Genome-wide multi-omics profiling of colorectal cancer identifies immune determinants strongly associated with relapse
Source: Front Genet. 2013 Nov 20;4:236. doi: 10.3389/fgene.2013.00236 (PMC3834519; doi:10.3389/fgene.2013.00236)
Supplement: Supplementary file 1 [file DataSheet1.ZIP › 66002_Madhavan_Data_Sheet_3.DOCX]

**A B**


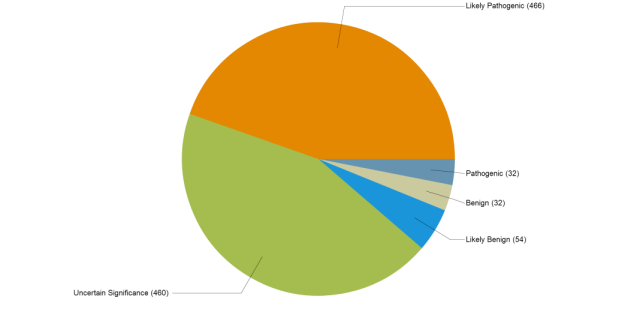

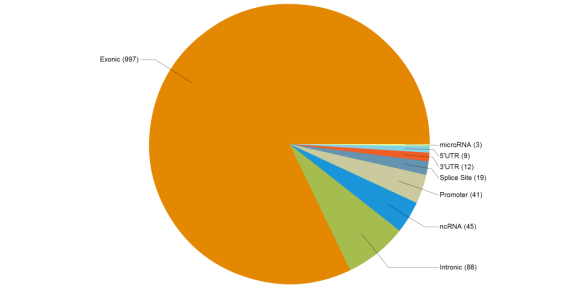


**Supplementary Figure 3: Variants by known impact and gene regions**

**(A)** Variants by known impact; **(B)** variants by gene regions
