# Supplementary material for: Genome-wide multi-omics profiling of colorectal cancer identifies immune determinants strongly associated with relapse
Source: Front Genet. 2013 Nov 20;4:236. doi: 10.3389/fgene.2013.00236 (PMC3834519; doi:10.3389/fgene.2013.00236)
Supplement: Supplementary file 1 [file DataSheet1.ZIP › 66002_Madhavan_Data_Sheet_2.DOCX]

## Supplemental file: Clinical data analysis

We applied two types of analysis – Kaplan Meier survival plots and Cox regression analysis. Kaplan Meier survival plots were done using <http://www.medcalc.org/> software to see how the clinical attributes affected survival. This analysis was done only on a few clinical variables namely – gender, tumor stage, tumor grade and vital status. Significance was computed using Log-rank test. P-values from the log-rank test and hazard ratios are shown in table above the KM plot.

Since KM plots cannot be applied to multiple input variables, we applied Cox regression analysis. Cox regression analysis (backward, stepwise) was done –using relapse status (i.e. outcome) as an endpoint and “**time from surgery to relapse or last follow up (FU)”** as survival time –to see which other clinical attributes had a significant effect on outcome and which clinical variables were retained in the final model. Various subsets of clinical data were applied as input to the Cox model. The significance level from the ‘Overall Model Fit’ shows the overall p-value of the model. Individual p-values from each covariate are shown in column “P” in the tables “Coefficients and Standard Errors.” The sample sizes in each analysis may be less than 40 because not all samples have data on all clinical variables. Only samples that have complete data on the clinical variables in each analysis are considered. Thus, all 40 sample data were input into each analysis but the model filtered out those samples that had missing values for any clinical attribute.

## KM plot with gender

| Survival time | SURGERY_TORECUR_FU_DAYS (Number of days from surgery to relapse or last follow up) |
| --- | --- |
| Endpoint | RECURRENCE_ANY (Relapse status) |
| Factor codes | GENDER |

| Comparison of survival curves (Logrank test) |  |  |
| --- | --- | --- |
| Endpoint: Observed n | 14.0 | 6.0 |
| Expected n | 9.7 | 10.3 |
| Chi-square | 3.8077 |  |
| DF | 1 |  |
| Significance | P = 0.0510 |  |

| Hazard ratio | 0.4006 |
| --- | --- |
| 95% CI | 0.1666 to 0.9629 |


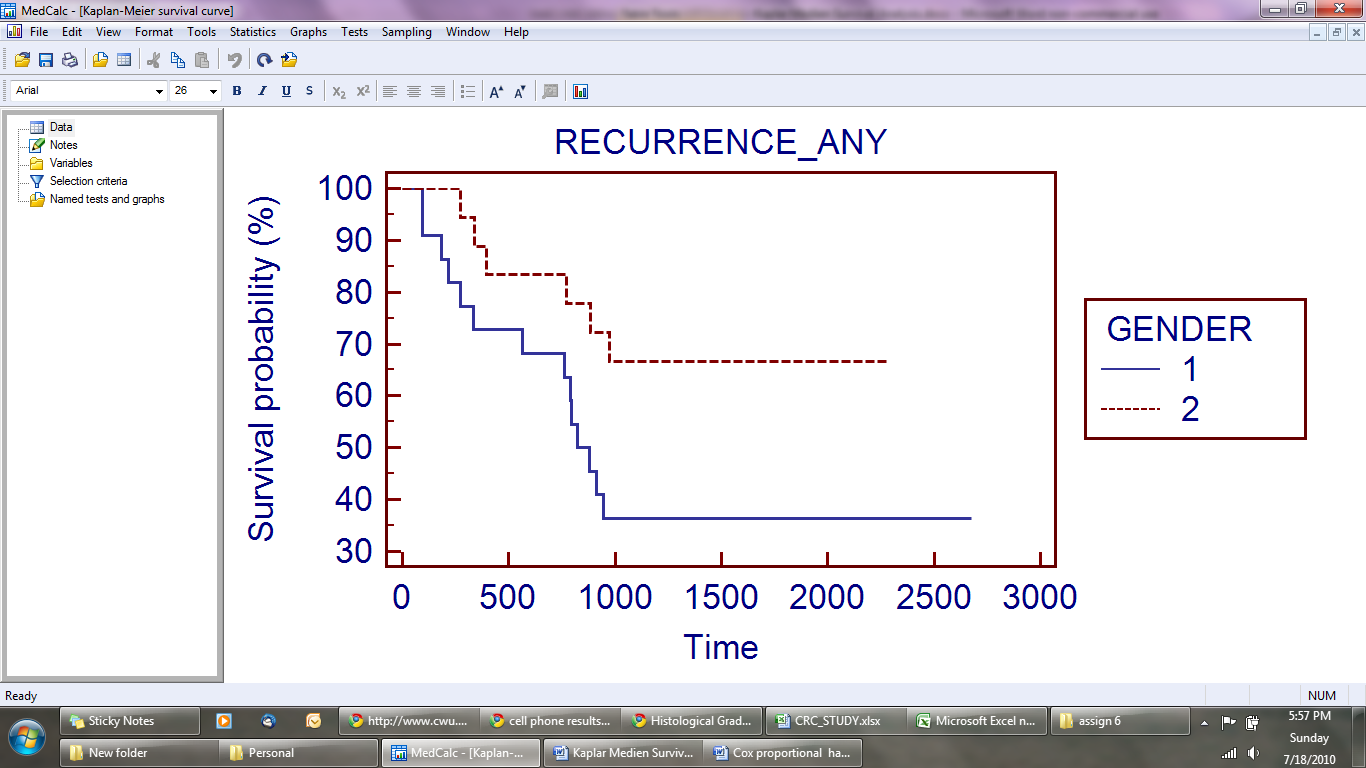


**Gender 1: Male**

**Gender 2: Female**

## KM plot with ‘Tumor Stage’

| Survival time | SURGERY_TORECUR_FU_DAYS (Number of days from surgery to relapse or last follow up) |
| --- | --- |
| Endpoint | RECURRENCE_ANY_0_1 (Relapse status) |
| Factor codes | TUMOR_STAGE (PTNM Tumor stage) |
|  |  |
|  |  |
|  |  |

|  | Comparison of survival curves (Logrank test) | | |
| --- | --- | --- | --- |
| Endpoint: Observed n | **4.0** | **12.0** | **4.0** |
| Expected n | 7.2 | 12.0 | 0.9 |
| Chi-square | 12.4858 |  | |
| DF | 2 |  | |
| Significance | P = 0.0019 |  | |


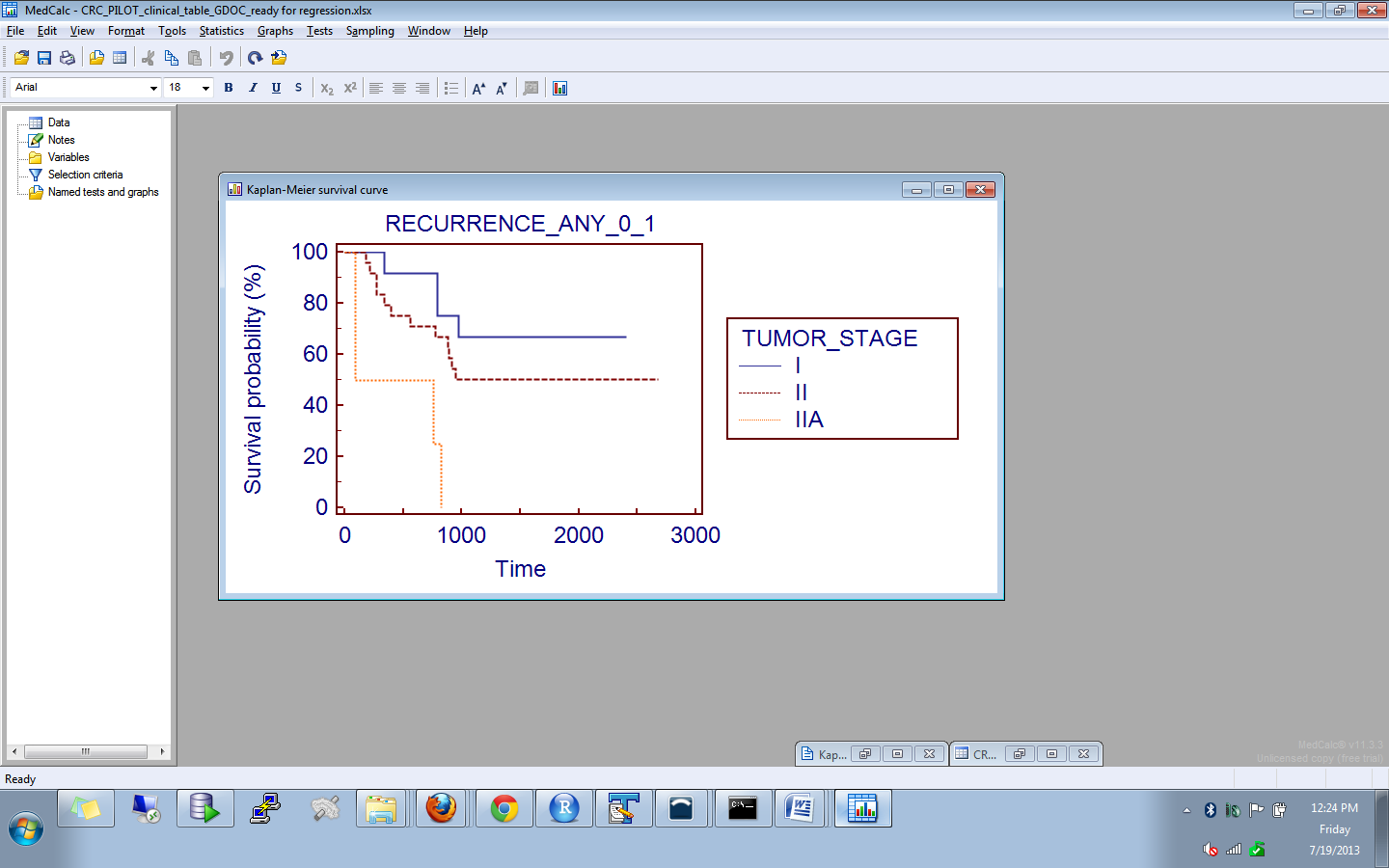


## KM plot with ‘Vital status’

| Survival time | SURGERY_TORECUR_FU_DAYS (Number of days from surgery to relapse or last follow up) | | |
| --- | --- | --- | --- |
| Endpoint | RECURRENCE_ANY (Relapse status) | | |
| Factor codes | VITAL_STATUS (Vital Status) | | |
|  | |  | |
|  | |  | |
|  | |  |  |
|  | |  |  |
|  | |  |  |
|  | |  |  |
|  | |  |  |

|  | Comparison of survival curves (Logrank test) | |
| --- | --- | --- |
| Endpoint: Observed n | 16.0 | 4.0 |
| Expected n | 18.9 | 1.1 |
| Chi-square | 8.3152 |  |
| DF | 1 |  |
| Significance | P = 0.0039 |  |

| Hazard ratio | 4.2998 |
| --- | --- |
| 95% CI | 0.6283 to 29.4251 |


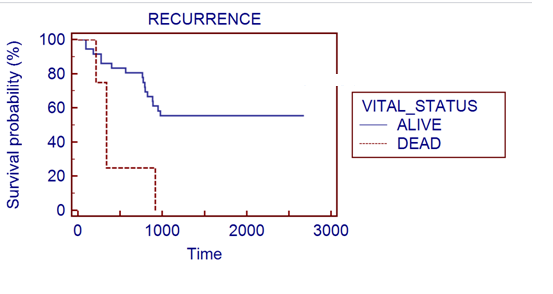


## KM plot with tumor grade

| Survival time | SURGERY_TORECUR_FU_DAYS (Number of days from surgery to relapse or last follow up) |
| --- | --- |
| Endpoint | RECURRENCE_ANY_0_1 (Relapse status) |
| Factor codes | TUMOR_GRADE (tumor grade) |

|  | Comparison of survival curves (Logrank test) | |
| --- | --- | --- |
| Endpoint: Observed n | 15.0 | 5.0 |
| Expected n | 17.5 | 2.5 |
| Chi-square | 2.7714 |  |
| DF | 1 |  |
| Significance | P = 0.0960 |  |

| Hazard ratio | 2.2984 |
| --- | --- |
| 95% CI | 0.6154 to 8.5843 |


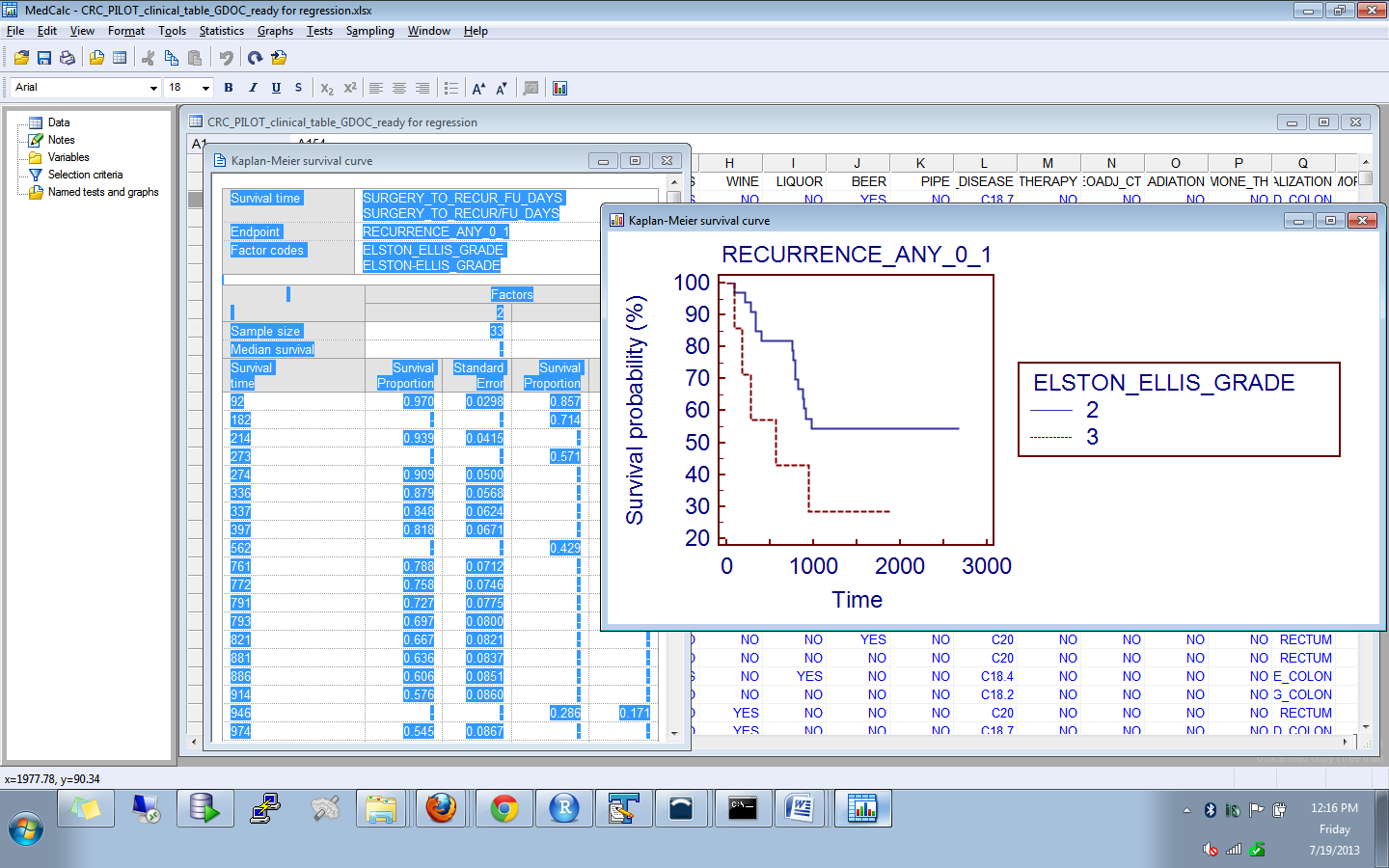


TUMOR GRADE

Grade 2

Grade 3

## Cox proportional-hazards regression analysis – 1

| Survival time | SURGERY_TO_RECUR_FU_DAYS (Number of days from surgery to relapse or last follow up) |
| --- | --- |
| Endpoint | RECURRENCE_ANY (Relapse status) |
| Method | Backward |
| Enter variable if P< | 0.05 |
| Remove variable if P> | 0.1 |
| Sample size | 24 |

**Overall Model Fit**

| Null model -2 Log Likelihood | 64.465 |
| --- | --- |
| Full model -2 Log Likelihood | 31.671 |
| Chi-square | 32.794 |
| DF | 11 |
| Significance level | P = 0.0006 |

**Coefficients and Standard Errors**

| Covariate | b | SE | P | Exp(b) | 95% CI of Exp(b) |
| --- | --- | --- | --- | --- | --- |
| bilirubin_[mg_dl] | 81.9094 | 33.8737 | 0.0156 | 374E+033 | 7692369.7721 to 18.2E+063 |
| cholesterol_[mg_dl] | 82.2953 | 32.3948 | 0.0111 | 550E+033 | 202318359.2279 to 1.50E+063 |
| creatinine_[mg_dl] | 13.7009 | 6.6169 | 0.0384 | 891744.3661 | 2.2210 to 358E+009 |
| CRP_[mg_l] | 0.6115 | 0.2434 | 0.0120 | 1.8432 | 1.1468 to 2.9625 |
| glucose_[mg_dl] | -0.7449 | 0.3264 | 0.0225 | 0.4748 | 0.2513 to 0.8973 |
| HDL_cholesterol_[mg_dl] | -82.9885 | 32.6945 | 0.0111 | 0.0000 | 0.0000 to 0.0000 |
| LDL_cholesterol_[mg_dl] | -82.3157 | 32.3974 | 0.0111 | 0.0000 | 0.0000 to 0.0000 |
| PTT_[sec.] | -9.7484 | 3.8028 | 0.0104 | 0.0001 | 0.0000 to 0.0970 |
| quick_test_[%] | 2.3380 | 0.9048 | 0.0098 | 10.3604 | 1.7747 to 60.4810 |
| triglycerides_[mg_dl] | -16.1259 | 6.3452 | 0.0110 | 0.0000 | 0.0000 to 0.0235 |
| uric_acid_[mg_dl] | -6.5307 | 2.6134 | 0.0125 | 0.0015 | 0.0000 to 0.2382 |

| Variables not included in the model |
| --- |
| alcaline_phosphatase_[U_l] |
| carbamide_[mg_dl] |
| CHE_[U_l] |
| GPT_[U_l] |
| LDH_[U_l] |
| potassium_[mMol_l] |

## Cox proportional-hazards regression analysis- 2

| Survival time | SURGERY_TO_RECUR_FU_DAYS (Number of days from surgery to relapse or last follow up) |
| --- | --- |
| Endpoint | RECURRENCE_ANY (Relapse status) |
| Method | Stepwise |
| Enter variable if P< | 0.05 |
| Remove variable if P> | 0.1 |
| Sample size | 40 |

**Overall Model Fit**

| Null model -2 Log Likelihood | 136.021 |
| --- | --- |
| Full model -2 Log Likelihood | 133.028 |
| Chi-square | 2.993 |
| DF | 1 |
| Significance level | P = 0.0836 |

**Coefficients and Standard Errors**

| Covariate | b | SE | P | Exp(b) | 95% CI of Exp(b) |
| --- | --- | --- | --- | --- | --- |
| DISEASE_LOCALIZATION="TRANSVERSE_COLON" | 1.6278 | 0.7945 | 0.0405 | 5.0926 | 1.0816 to 23.9775 |

| Variables not included in the model |
| --- |
| AGE_CASE_START |
| BEER="YES" |
| BMI_GROUP="OBESE" |
| BMI_GROUP="OVERWEIGHT" |
| BMI_GROUP="UNDERWEIGHT" |
| CIGARETTES="YES" |
| DISEASE_LOCALIZATION="ASCENDING_COLON" |
| DISEASE_LOCALIZATION="ILEOCAECAL" |
| DISEASE_LOCALIZATION="LEFT_FLEXURE" |
| DISEASE_LOCALIZATION="SIGMOID_COLON" |
| TUMOR_GRADE=3 |
| GENDER="FEMALE" |

## Cox proportional-hazards regression analysis - 3

| Survival time | SURGERY_TO_RECUR_FU_DAYS (Number of days from surgery to relapse or last follow up) |
| --- | --- |
| Endpoint | RECURRENCE_ANY (Relapse status) |
| Method | Stepwise |
| Enter variable if P< | 0.05 |
| Remove variable if P> | 0.1 |
| Sample size | 26 |

**Overall Model Fit**

| Null model -2 Log Likelihood | 77.419 |
| --- | --- |
| Full model -2 Log Likelihood | 73.936 |
| Chi-square | 3.483 |
| DF | 1 |
| Significance level | P = 0.0620 |

**Coefficients and Standard Errors**

| Covariate | b | SE | P | Exp(b) | 95% CI of Exp(b) |
| --- | --- | --- | --- | --- | --- |
| BMI_GROUP="OBESE" | 1.1503 | 0.5807 | 0.0476 | 3.1591 | 1.0180 to 9.8029 |

| Variables not included in the model |
| --- |
| bilirubin_[mg_dl] |
| BMI_GROUP="IDEAL" |
| BMI_GROUP="UNDERWEIGHT" |
| cholesterol_[mg_dl] |
| creatinine_[mg_dl] |
| DISEASE_LOCALIZATION="ASCENDING_COLON" |
| DISEASE_LOCALIZATION="ILEOCAECAL" |
| DISEASE_LOCALIZATION="LEFT_FLEXURE" |
| DISEASE_LOCALIZATION="SIGMOID_COLON" |
| triglycerides_[mg_dl] |

## Conclusion:

This analysis shows us that tumor stage and other variables related to clinical chemistry parameters –Glucose, Bilirubin, Creatinine, CRP, and triglycerides may be putatively associated with relapse status and survival. Some of the confidence intervals of the hazard ratios are extremely large and indicate that they are not stable. Many of the clinical variables known to be linked with colon cancer such as use of alcohol, tumor grade, BMI and gender did not show significant association with outcome. Hence, the use of clinical data alone with low sample size does not give enough predictive power. We did not do any further analysis with clinical data (no model adjustment was done), and investigated the genomic data to see if it had better predictive power.
