# Supplementary material for: Genome-wide multi-omics profiling of colorectal cancer identifies immune determinants strongly associated with relapse
Source: Front Genet. 2013 Nov 20;4:236. doi: 10.3389/fgene.2013.00236 (PMC3834519; doi:10.3389/fgene.2013.00236)
Supplement: Supplementary file 1 [file DataSheet1.ZIP › 66002_Madhavan_Data_Sheet_4.DOCX]

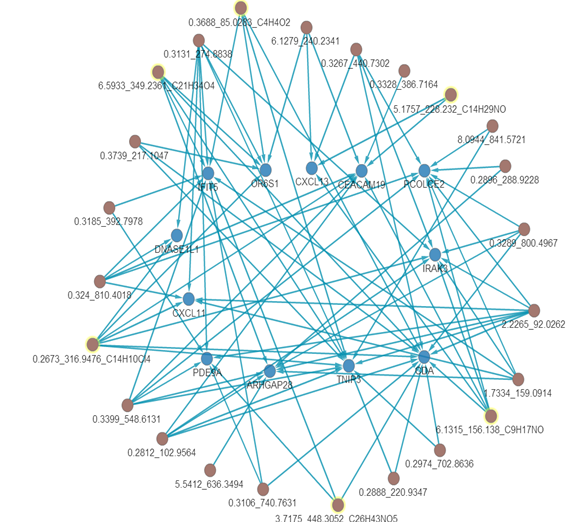

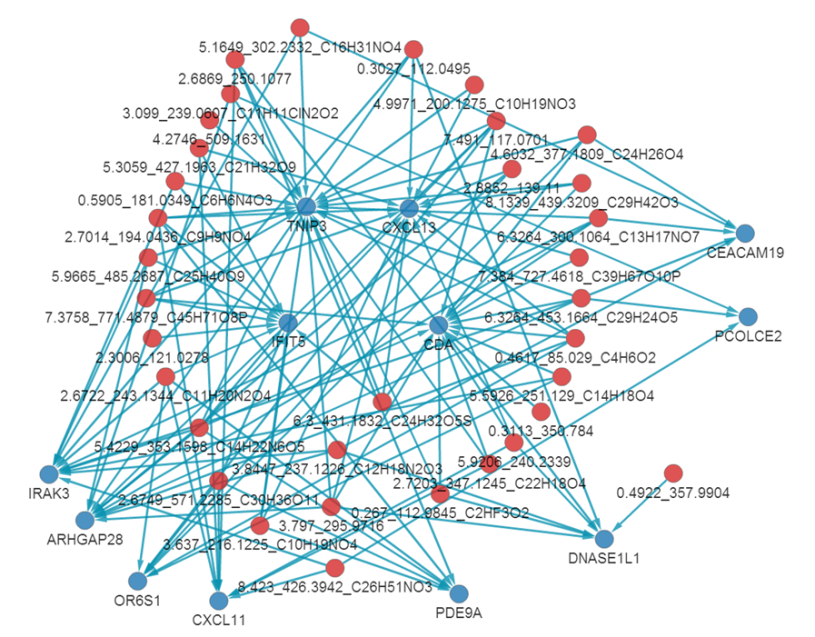

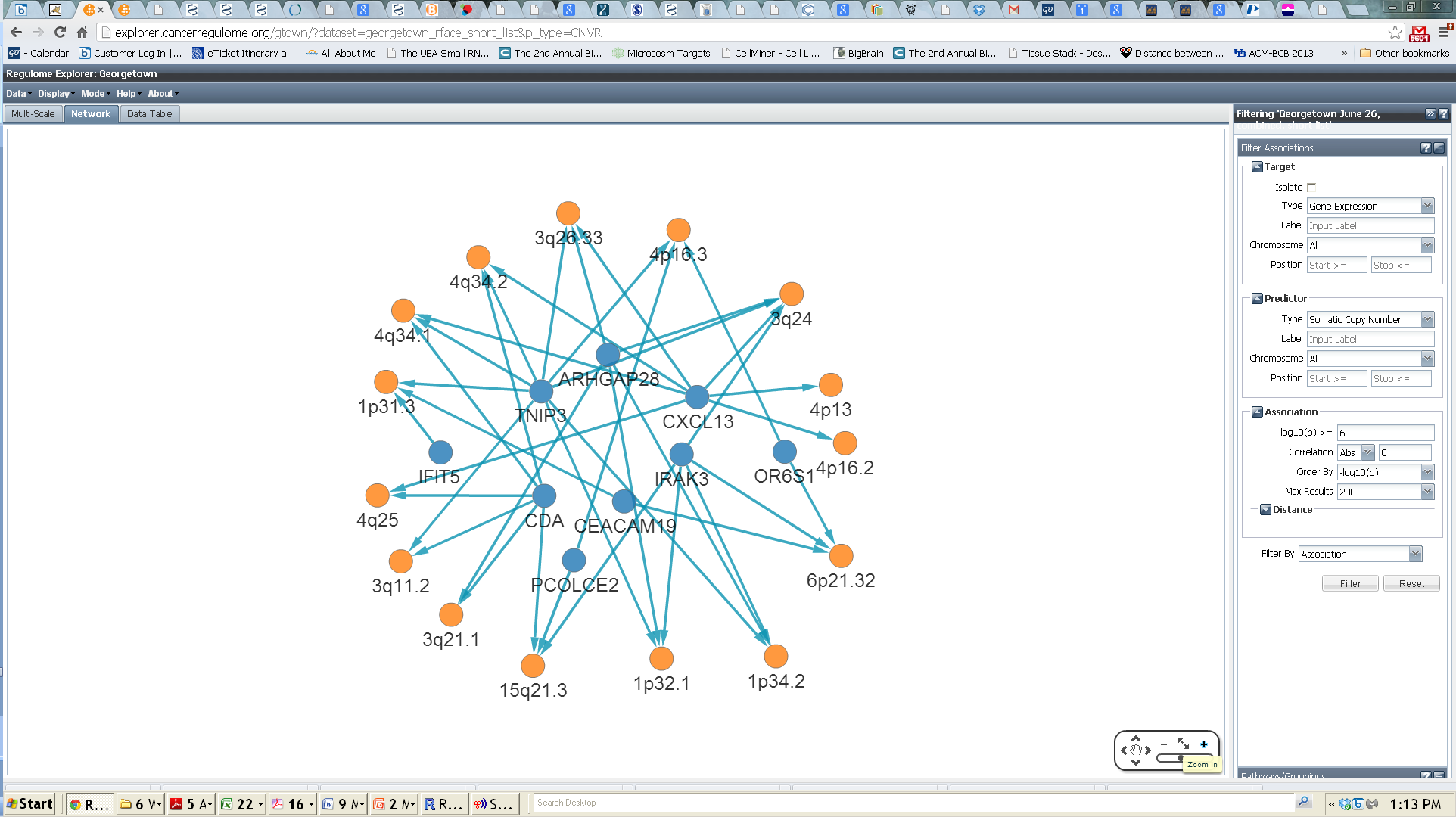

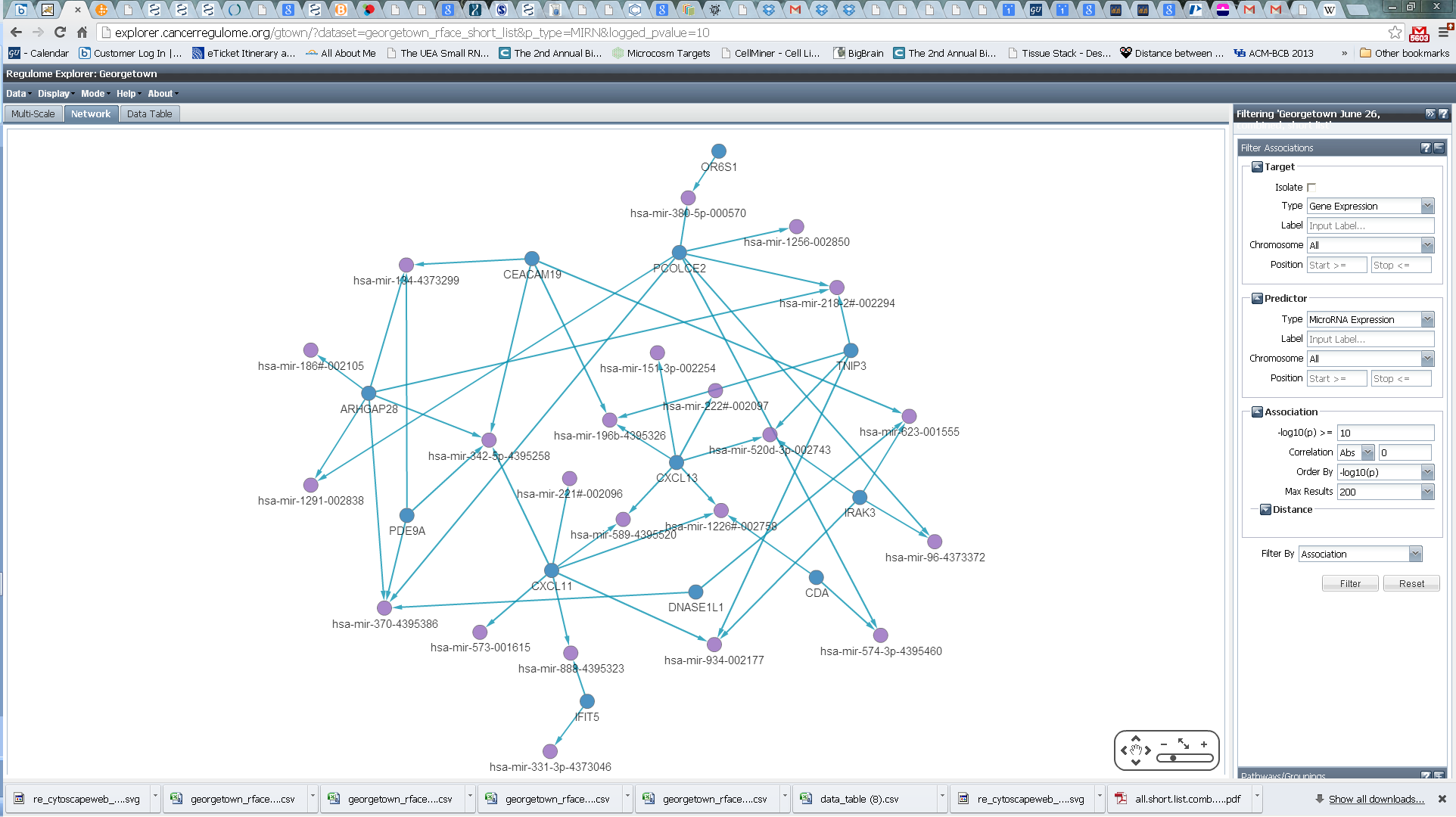


A

B

D

C

**Supplementary Figure 4: Sub networks with two data types**

Subnetworks showing associations between two pre-selected data types. **(A)** Predictors - genes; Target – serum metabolites. **(B)** Predictors - genes; Target – urine metabolites. **(C)** Predictor - CNV; Target – Genes. **(D)** Predictor: miRNA; Target: Genes.
